# Auxin-mediated regulation of susceptibility to toxic metabolites, c-di-GMP levels, and phage infection in the rhizobacterium *Serratia plymuthica*

**DOI:** 10.1128/msystems.00165-24

**Published:** 2024-06-05

**Authors:** Miriam Rico-Jiménez, Zulema Udaondo, Tino Krell, Miguel A. Matilla

**Affiliations:** 1 Department of Biotechnology and Environmental Protection, Estación Experimental del Zaidín, Consejo Superior de Investigaciones Científicas, Granada, Spain; 2 Department of Biomedical Informatics, University of Arkansas for Medical Sciences, Little Rock, Arkansas, Spain; Leiden University, Leiden, the Netherlands

**Keywords:** Bacteria, transcriptomics, signaling, auxin, biofilm, c-di-GMP, antibiotic susceptibility, phage sensitivity, indole-3-acetic acid, resistance to toxic metabolites

## Abstract

The communication between plants and their microbiota is highly dynamic and involves a complex network of signal molecules. Among them, the auxin indole-3-acetic acid (IAA) is a critical phytohormone that not only regulates plant growth and development, but is emerging as an important inter- and intra-kingdom signal that modulates many bacterial processes that are important during interaction with their plant hosts. However, the corresponding signaling cascades remain largely unknown. Here, we advance our understanding of the largely unknown mechanisms by which IAA carries out its regulatory functions in plant-associated bacteria. We showed that IAA caused important changes in the global transcriptome of the rhizobacterium *Serratia plymuthica* and multidisciplinary approaches revealed that IAA sensing interferes with the signaling mediated by other pivotal plant-derived signals such as amino acids and 4-hydroxybenzoic acid. Exposure to IAA caused large alterations in the transcript levels of genes involved in amino acid metabolism, resulting in significant metabolic alterations. IAA treatment also increased resistance to toxic aromatic compounds through the induction of the AaeXAB pump, which also confers resistance to IAA. Furthermore, IAA promoted motility and severely inhibited biofilm formation; phenotypes that were associated with decreased c-di-GMP levels and capsule production. IAA increased capsule gene expression and enhanced bacterial sensitivity to a capsule-dependent phage. Additionally, IAA induced the expression of several genes involved in antibiotic resistance and led to changes in the susceptibility and responses to antibiotics with different mechanisms of action. Collectively, our study illustrates the complexity of IAA-mediated signaling in plant-associated bacteria.

**IMPORTANCE:**

Signal sensing plays an important role in bacterial adaptation to ecological niches and hosts. This communication appears to be particularly important in plant-associated bacteria since they possess a large number of signal transduction systems that respond to a wide diversity of chemical, physical, and biological stimuli. IAA is emerging as a key inter- and intra-kingdom signal molecule that regulates a variety of bacterial processes. However, despite the extensive knowledge of the IAA-mediated regulatory mechanisms in plants, IAA signaling in bacteria remains largely unknown. Here, we provide insight into the diversity of mechanisms by which IAA regulates primary and secondary metabolism, biofilm formation, motility, antibiotic susceptibility, and phage sensitivity in a biocontrol rhizobacterium. This work has important implications for our understanding of bacterial ecology in plant environments and for the biotechnological and clinical applications of IAA, as well as related molecules.

## INTRODUCTION

The sensing of environmental signals allows bacteria to adapt to changes that occur in their ecological niches or during host colonization (1). The number of signal transduction systems encoded in the genomes of microorganisms with a complex lifestyle is particularly high (2, 3), suggesting that these microbes respond to a particularly broad range of physical, chemical, and biological stimuli. In this context, plants and their associated microorganims are holobionts (4–6), and the interaction between plants and their microbiota, as well as between different phyto-microorganisms, is highly dynamic and controlled by the concerted action of multiple signal molecules (1, 4, 7). This intricate communication shapes the structure of the plant microbiota (4, 6, 8).

The chemical diversity of plant signals that modulate bacterial physiology and metabolism is high, including amino and organic acids, sugars, peptides, aromatic acids, polyamines, phenolic compounds, fatty acids, phytohormones, inorganic ions, and volatiles (1, 4, 7, 9–12). Given the complexity of this chemical language, plant-associated bacteria (PAB) possess multiple mechanisms that permit the generation of responses to the signals sensed (1, 3, 7, 13). For example, the important role of plant-bacteria communication is reflected by the fact that PAB have, on average, twice as many chemoreceptors compared to bacteria that do not interact with plants (2). Nonetheless, the molecular mechanisms by which this plant-bacteria communication occurs remain largely unknown (6, 7).

A number of key plant signaling molecules were shown to be of great relevance to establish plant-bacteria interactions, including cytokinins (7, 14), ethylene (15), gibberelins (16, 17), salicylic acid (1, 15), abscisic acid (15, 18), auxins (19, 20), ɣ-aminobutyric acid (7, 21), and strigolactones (15, 22). The auxin indole-3-acetic acid (IAA) is a key phytohormone that coordinates plant growth and development, as well as plant responses to a broad range of biotic and abiotic stresses (23–25). In addition, IAA is an essential signal molecule in the interaction between plants and microbes. Thus, next to its production by plants, most PAB were found to produce significant amounts of IAA (26, 27), indicating that IAA-mediated plant-bacteria communication is bidirectional and as such highly complex. IAA production by beneficial PAB typically results in plant growth promotion (15, 19), but alterations in IAA homeostasis in the plant by phytopathogenic bacteria are closely associated with virulence-related processes such as tumor formation, epiphytic colonization, or modulation of plant defenses (20).

In addition to its role as an inter-kingdom signal, IAA is an important bacterial signal molecule that regulates biofilm formation, primary and secondary metabolism, auxin catabolism, virulence factor production, chemotactic responses, and plant host colonization, among other processes (19, 20, 28–33). However, the molecular mechanisms underlying these regulatory processes have been identified in only a few cases, such as the IAA-mediated virulence suppression through the inhibition of phytotoxin biosynthesis (28). Likewise, the regulation of the IAA catabolism depended on several IAA-responsive transcriptional regulators (30). Additionally, we have recently identified a chemoreceptor that mediates IAA chemoattraction in a beneficial PAB (31). Notably, bacterial signal transduction receptors can also be stimulated by the binding of signal-loaded solute-binding proteins (13) and several IAA-responsive solute-binding proteins have been identified (32, 34) that may potentially exert a role in signal transduction.

We have recently established the rhizosphere biocontrol agent *Serratia plymuthica* A153 (35) as a model microorganism in the study of IAA-mediated signaling. Initial studies revealed that the LysR-type transcriptional regulator (LTTR) AdmX acts as an activator of the synthesis of the antibiotic andrimid in A153 (36). We subsequently showed that the ligand-binding domain (LBD) of AdmX recognizes IAA and its biosynthetic intermediate indole-3-pyruvic acid. Whereas IAA binding regulated both andrimid production and the expression of the corresponding biosynthetic gene cluster in A153, indole-3-pyruvic acid did not exert a regulatory role (37)—highlighting the biological relevance of IAA over other structurally similar auxins. Moreover, we also demonstrated that auxin-responsive LBDs have specifically emerged in PAB (38), illustrating the existence of bacterial receptors that specifically respond to plant-derived compounds.

The inactivation of IAA synthesis resulted in important transcriptional changes in *S. plymuthica* A153 (39), supporting that IAA has signaling functions in this rhizobacterium. This, together with the fact that A153 cannot catabolize IAA (37), makes it an excellent model to study the signaling roles of IAA. Here, we show that IAA caused major changes in the global transcriptome of *S. plymuthica* and investigated the mechanisms by which this auxin modulated a variety of metabolic and physiological features.

## RESULTS

### IAA treatment causes important changes in transcript levels

**Fig 1:**
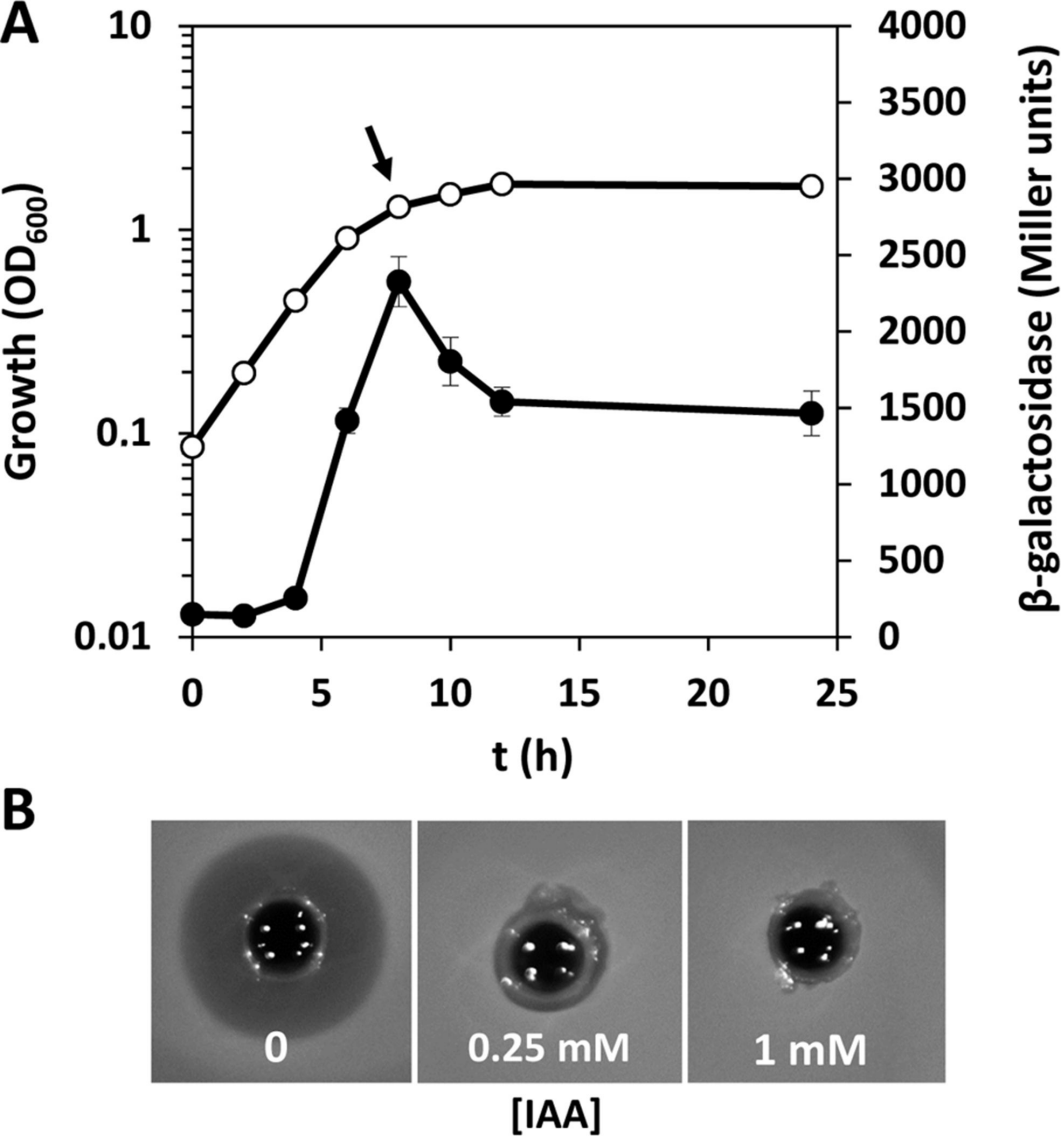
The expression profile of the andrimid biosynthetic gene cluster of *Serratia plymuthica* A153 and sample collection for the transcriptomics assays. (**A**) Transcription of the andrimid biosynthetic cluster measured from a chromosomal fusion *admK::lacZ* in *S. plymuthica* A153. Growth (open symbols) and β-galactosidase activity (filled symbols) were measured in minimal medium supplemented with 15 mM glucose at 25°C. Data are the means and standard deviations of three biological replicates. Some standard deviations are minor and are not visible in the corresponding growth curves. Arrow, time point when samples for RNA-seq were taken. (**B**) Andrimid production by *S. plymuthica* A153 under the experimental conditions in which RNA-seq samples were taken. For the assays, a *Bacillus subtilis* top agar lawn was prepared and 300 µL of filter-sterilized supernatants were added to holes punched in the *Bacillus* bioassay plates.

To investigate the effects of IAA on the global transcriptome of *S. plymuthica* A153, we used transcriptome sequencing (RNA-seq). For sample preparation, A153 was grown in minimal medium with glucose as sole carbon source in the absence and presence of 0.25 and 1 mM IAA. At these IAA concentrations, this auxin had no effect on the growth rate, as evidenced by the count of colony-forming units (Fig. S1). Samples for RNA-seq were collected at the beginning of the stationary phase; stage at which the expression of the andrimid gene cluster was maximal (Fig. 1A). Analysis of the antibiotic activity of the culture supernatants showed a near or complete loss of andrimid production in the presence of 0.25 and 1 mM IAA, respectively (Fig. 1B).

**Fig 2:**
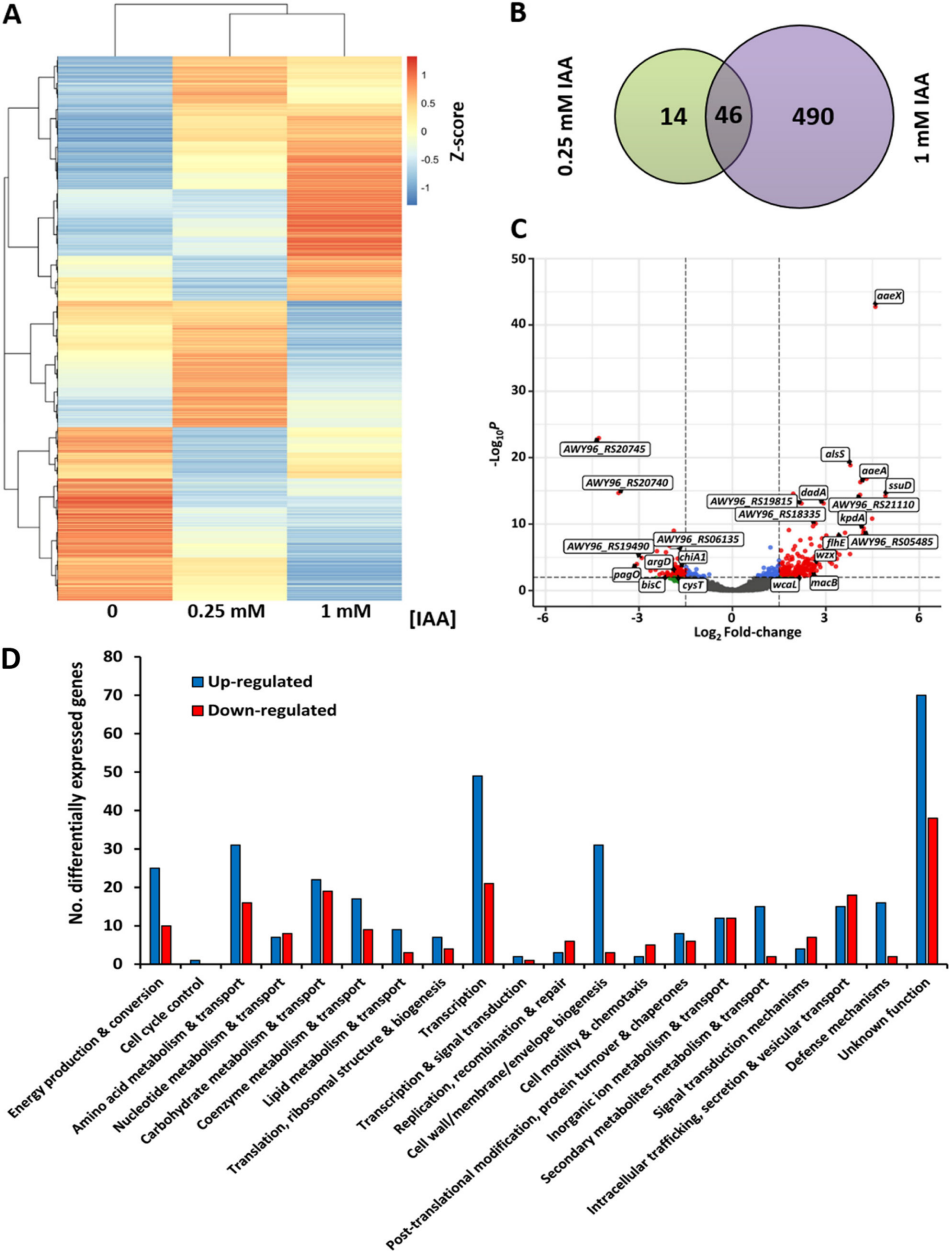
The transcriptome of *Serratia plymuthica* A153 in response to indole-3-acetic acid (IAA). (**A**) Heatmaps derived from A153 growth in minimal medium supplemented with 15 mM glucose in the absence and presence of 0.25 and 1 mM IAA. (**B**) Venn diagram showing the number of differentially expressed genes (log_2_ fold change ≥|1.5| and an adjusted *P*-value inferior to 0.05) in the presence of 0.25 mM and 1 mM IAA. (**C**) Volcano plot of differentially transcribed genes in response to 1 mM IAA. The log_2_ (fold change) was plotted against the statistical significance (−log_10_ of the adjusted *P*-value < 0.05) for each gene. Vertical dashed lines represent the log_2_ fold change cut-off of ≥|1.5|. Red dots represent significant differentially expressed genes. (**D**) Functional classification of the differentially regulated genes in response to 1 mM IAA. Functional categories were defined according to clusters of orthologous genes (COGs). For clarity, some categories according to COGs were combined, as specified in Table S1.

RNA-seq data revealed that IAA treatment caused important transcriptional changes in A153. Statistically significant differentially expressed genes (DEGs) were selected based on a fold-change magnitude of log_2_ greater than 1.5 and an adjusted *P*-value (*p*adj) inferior to 0.05, according to Benjamini and Hochberg’s approach (40). A total of 60 and 536 DEGs were identified in response to 0.25 and 1 mM IAA, respectively, which correspond to 1.2% and 13.2% of the A153 genes (Fig. 2; Tables S1 and S2). Whereas the number of induced and repressed genes were balanced in response to 0.25 mM IAA (34 and 26 DEGs up- and downregulated, respectively), a preference for gene induction (346 and 190 DEGs up- and downregulated, respectively) was observed in response to 1 mM IAA (Fig. 2; Table 1; Tables S1 and S2). Notably, ~78% of the DEGs identified in response to 0.25 mM IAA were also differentially expressed in response to 1 mM IAA (Fig. 2B; Table S3). In addition, our RNA-seq data also allowed to identify 273 sRNAs, of an average length of 104 bp, that were all located in intergenic regions (Table S4). The analysis of the transcript levels of these sRNAs revealed that 51 (24 and 27 up- and downregulated, respectively) and 50 (35 and 15 up- and downregulated, respectively) were differentially expressed in response to 0.25 mM and 1 mM IAA, respectively (Table S4). Given the markedly larger number of transcriptional changes caused by 1 mM IAA (Fig. 2; Tables S1-S4), some of our phenotypical analyses were primarily centered on this condition.

**TABLE 1:** Selected differentially expressed genes regulated in response to 1 mM indole-3-acetic acid^*a*^

Locus no.	Preferred gene name	Known or predicted function	Log_2_ fold change^*b*^	COG class^*c*^
Upregulated				
Energy production and conversion				
AWY96_RS01055	*ssuD*	FMNH_2_-dependent alkanesulfonate monooxygenase	6.0	C
AWY96_RS06475^*d*^	*mdaB*	NADPH quinone reductase	3.1	S
AWY96_RS12020^*d*^	*ywrO*	NADPH-dependent FMN reductase	4.3	S
Amino acid transport and metabolism				
AWY96_RS02860	*alsS*	Acetolactate synthase	4.1	EH
AWY96_RS05925	*proC*	Pyrroline-5-carboxylate reductase. L-Pro metabolism	2.0	E
AWY96_RS08640	*gdhA*	Glutamate/phenylalanine/leucine/valine/L-tryptophandehydrogenase	1.6	E
AWY96_RS09110^*d*^	*–*	LysE family transporter	1.9	E
AWY96_RS12990	*yjjB*	Threonine/serine exporter	3.8	E
AWY96_RS22590^*d*^	*trpE*	Anthranilate synthase component	2.5	E
AWY96_RS22970	*dadA*	D-amino acid dehydrogenase	3.1	E
Carbohydrate metabolism and transport				
AWY96_RS05485	*–*	Putative sugar transporter	5.4	G
AWY96_RS16915	*–*	Phosphoenolpyruvate phosphomutase	4.6	G
AWY96_RS21140^*d*^	*–*	Carbohydrate periplasmic-binding protein	5.5	G
AWY96_RS23630	*–*	Glycosyl transferase	3.2	G
Transcription				
AWY96_RS01340	*–*	Transcriptional regulator	1.9	K
AWY96_RS01440	*–*	LysR type transcriptional regulator	3.4	K
AWY96_RS02175	*lrhA*	LysR type transcriptional regulator	1.9	K
AWY96_RS14165	–	TetR type transcriptional regulator	4.4	K
AWY96_RS16235	*ycfQ*	TetR type transcriptional regulator	2.2	K
AWY96_RS16810	*–*	LuxR type transcriptional regulator	1.8	KT
AWY96_RS21015	*–*	LysR type transcriptional regulator	3.1	K
Cell wall/membrane/envelop biogenesis				
AWY96_RS01950	*ompC*	Porin	2.1	M
AWY96_RS17105	*galU*	UTP-glucose-1-phosphate uridylyltransferase	1.9	M
AWY96_RS17115	*wza*	Polysaccharide export protein	1.9	M
AWY96_RS17130	*wcaK*	Pyruvyl transferase	2.2	S
AWY96_RS17135	*wcaL*	Glycosyl transferase	2.5	M
AWY96_RS17140	*amsL*	Glycosyl transferase	2.3	S
AWY96_RS17145	*wzx*	Glycosyl transferase	3.1	G
AWY96_RS17150	*–*	Hypothetical protein	2.0	–
AWY96_RS17155	*rfbU*	Glycosyl transferase	2.7	M
AWY96_RS17160	*–*	Glycosyl transferase	2.8	M
AWY96_RS17165	*rfbP*	Bacterial sugar transferase	2.5	M
AWY96_RS17170	–	Right-handed parallel beta-helix repeat-containing protein	2.8	–
AWY96_RS17175	*–*	Hypothetical protein	2.3	Q
AWY96_RS17180	*prsD*	Secretion system permease	1.7	V
AWY96_RS17195	*cpsB*	Mannose-6-phosphate isomerase	1.5	GM
AWY96_RS17200	*manB*	Phosphoglucomutase/phosphomannomutase	1.9	G
AWY96_RS17205	*galE*	UDP-glucose 4-epimerase	1.6	M
AWY96_RS17240	*wbpY*	Glycosyl transferase	1.8	M
Cell motility				
AWY96_RS24070	*flhE*	Flagellar protein	4.0	N
Inorganic ion transport and metabolism				
AWY96_RS02430^*d*^	*cusA*	Metal efflux RND transporter	3.7	P
AWY96_RS15400	*kpdB*	Potassium transport system	2.5	P
AWY96_RS15405	*kpdA*	Potassium transport system	5.2	P
AWY96_RS21110	–	TonB-dependent receptor	4.7	P
Secondary metabolites biosynthesis, transport, and catabolism				
AWY96_RS00650	*admN*	Dabb family protein	1.9	–
AWY96_RS00655	*admM*	Polyketide synthase	1.5	–
AWY96_RS00660	*admL*	Ester cyclase	1.9	–
AWY96_RS00665	*admK*	Non-ribosomal peptide synthetase	1.6	–
AWY96_RS00685	*admG*	Dehydratase	1.5	–
AWY96_RS00695	*admE*	Dehydratase	2.1	–
AWY96_RS00710	*admB*	Hypothetical protein	1.7	–
Signal transduction mechanisms				
AWY96_RS16815	*–*	Diguanylate phosphodiesterase—EAL domain-containing protein	2.0	T
AWY96_RS18985^*d*^	*–*	Diguanylate cyclase	1.8	T
AWY96_RS19815	–	Diguanylate phosphodiesterase—EAL domain-containing protein	2.3	T
Intacellular trafficking, secretion, and vesicular transport				
AWY96_RS03475	*mdtB*	Multidrug efflux RND transporter permease subunit	1.9	U
AWY96_RS07095^*d*^	*aaeB*	*p*-Hydroxybenzoic acid efflux pump subunit B	3.9	U
AWY96_RS07100^*d*^	*aaeA*	*p*-Hydroxybenzoic acid efflux pump subunit A	4.5	U
AWY96_RS07105	*aaeX*	Hypothetical protein	4.8	S
AWY96_RS21755	*mhbT*	*p*-Hydroxybenzoic acid transporter	2.1	G
AWY96_RS22460^*d*^	*cstA*	Carbon starvation protein CstA	1.6	T
Defense mechanisms				
AWY96_RS05000	*macB*	Macrolide ABC transporter permease	3.7	V
AWY96_RS08600	*srfA*	Effector protein	2.5	S
AWY96_RS12040	–	Coalicin immunity protein	2.5	S
AWY96_RS15045	–	Multidrug resistance protein	2.1	E
AWY96_RS17030^*d*^	*sanA*	Outer membrane permeability protein	2.5	S
AWY96_RS18945	*–*	Universal stress protein	2.4	T
AWY96_RS20405^*d*^	*ampC*	Beta lactamase	2.4	V
Unknown function				
AWY96_RS12305^*d*^	–	Hypothetical protein	1.6	–
AWY96_RS18335^*d*^	*–*	Hypothetical protein	2.9	–
AWY96_RS19515^*d*^	*–*	Hypothetical protein	4.1	–
AWY96_RS20345^*d*^	*–*	Hypothetical protein	3.3	–
Downregulated				
Biosynthesis of cofactors, prosthetic groups and carriers				
AWY96_RS01740	*torA*	Oxidoreductase	−2.5	C
AWY96_RS03110	*napA*	Nitrate reductase subunit	−2.5	C
AWY96_RS04860	*bisC*	Oxidoreductase	−2.7	C
AWY96_RS10760^*d*^	*glpA*	Glycerol-3-phosphate dehydrogenase	−1.6	C
Amino acid transport and metabolism				
AWY96_RS06135^*d*^	–	Aminotransferase	−2.2	E
AWY96_RS09810	*artM5*	Amino acid ABC transporter permease	−2.7	E
AWY96_RS15915	–	Amino acid permease	−2.3	E
AWY96_RS23480	*argD*	Aspartate/lysine aminotransferase	−2.2	E
Carbohydrate metabolism and transport				
AWY96_RS06350^*d*^	*scrB*	Sucrose-6-phosphate hydrolase	−2.4	G
AWY96_RS08090	*chbF*	Phospho-beta-glucosidase	−2.5	G
AWY96_RS20750	*pagO*	Transporter permease	−4.8	EG
AWY96_RS22870^*d*^	*chiA1*	Chitinase protein	−1.9	G
Transcription				
AWY96_RS00930	–	LysR type transcriptional regulator	−2.9	K
AWY96_RS04715	*gcvA*	LysR type transcriptional regulator	−1.7	K
AWY96_RS16860	–	TetR type transcriptional regulator	−1.7	K
AWY96_RS18445	–	AraC type transcriptional regulator	−2.3	K
AWY96_RS21070	*csoR*	Metal-sensing transcriptional regulator	−1.7	S
AWY96_RS21150	*ttdR*	LysR type transcriptional regulator	−2.1	K
Cell motility and secretion				
AWY96_RS08755	–	Fimbrial protein	−1.9	NU
AWY96_RS24110	*cheD*	Chemotaxis protein	−2.2	NT
AWY96_RS24125	*cheA*	Chemotaxis protein	−2.3	NT
Inorganic ion transport and metabolism				
AWY96_RS00935^*d*^	–	Periplasmic-binding protein	−2.1	P
AWY96_RS02710	*yfeO*	Ion-transport protein	−2.2	P
AWY96_RS02980	*cysT*	Sulfate/thiosulfate ABC transporter permease	−3.3	P
AWY96_RS13465	*cysJ*	Sulfite reductase flavoprotein subunit	−2.5	P
AWY96_RS19845	*hemR*	TonB-dependent receptor	−1.7	P
Signal transduction mechanisms				
AWY96_RS02945	*cpxA_1*	Histidine kinase	−1.9	T
AWY96_RS04360	*citA*	Histidine kinase	−1.7	T
AWY96_RS05735	*qseC*	Histidine kinase	−2.5	T
AWY96_RS15100	*adrA*	Diguanylate cyclase	−2.1	T
Intracellular trafficking, secretion, and vesicular transport				
AWY96_RS12630^*d*^	–	Major facilitator superfamily transporter	−2.0	EGP
AWY96_RS16165^*d*^	–	Major facilitator superfamily transporter	−2.0	EGP
AWY96_RS15555	*tolR*	Colicin uptake protein	−2.0	U
AWY96_RS20910	–	Major facilitator superfamily transporter	−3.5	EGP
Defense mechanisms				
AWY96_RS22230	*asr*	Acid resistance protein	−2.1	S
Unknown function				
AWY96_RS06140^*d*^	–	Hypothetical protein	−2.4	–
AWY96_RS14970^*d*^	–	Hypothetical protein	−2.1	–
AWY96_RS19490	–	Putative glutathione-dependent formaldehyde-activating enzyme	−3.8	S
AWY96_RS20740^*d*^	–	Hypothetical protein	−4.3	–
AWY96_RS20745^*d*^	–	Hypothetical protein	−4.8	–

^*a*^ The complete list of differentially expressed genes is provided in Table S1.

^*b*^ *P*-value adjusted lower than 0.05.

^*c*^ C, energy production and conversion; E, amino acid transport and metabolism; G, carbohydrate transport and metabolism; H, coenzyme transport and metabolism; K, transcription; M, cell wall/membrane/envelope biogenesis; N, cell motility; P, inorganic ion transport and metabolism; Q, secondary metabolites biosynthesis, transport and catabolism; S, function unknown; T, signal transduction mechanisms; U, intracellular trafficking, secretion, and vesicular transport; V, defense mechanisms.

^*d*^ Differentially expressed gene also identified in response to 0.25 mM indole-3-acetic acid treatment.

### Large functional diversity of IAA-regulated genes

Using clusters of orthologous genes (COGs) (41), DEGs were classified into 20 functional categories (Fig. 2D; Fig. S2; Tables S1 and S2), indicative of the complexity of the IAA-mediated responses. In response to 0.25 mM IAA, most populated categories were amino acid transport and metabolism (15%), carbohydrate metabolism and transport (8.3%), energy production and conversion (11.6%), transcription (6.7%), and inorganic transport and metabolism (8.3%) (Fig. S2; Table S2). Alternatively, amino acid transport and metabolism (8.8%), carbohydrate metabolism and transport (7.6%), energy production and conversion (6.5%), transcription (13.1%), coenzyme transport and metabolism (4.9%), cell wall/membrane biogenesis (6.3%), inorganic transport and metabolism (4.8%), and intracellular trafficking and secretion (6.2%) were the most populated functional categories in response to 1 mM IAA, with 20.2% of the DEGs being of unknown function (Fig. 2D; Table S1).

To validate our RNA-seq data, we performed quantitative real time PCR (RT-qPCR) assays of a selection of 14 DEGs belonging to 9 functional categories (e.g., metabolism and transport of organic and inorganic metabolites, cell wall and membrane biogenesis, transcription, signal transduction, and intracellular trafficking). The results correlated well with the RNA-seq data (Fig. S3; Tables S1 and S2).

### IAA treatment caused large alterations in the metabolic capabilities of *Serratia plymuthica*

**Fig 3:**
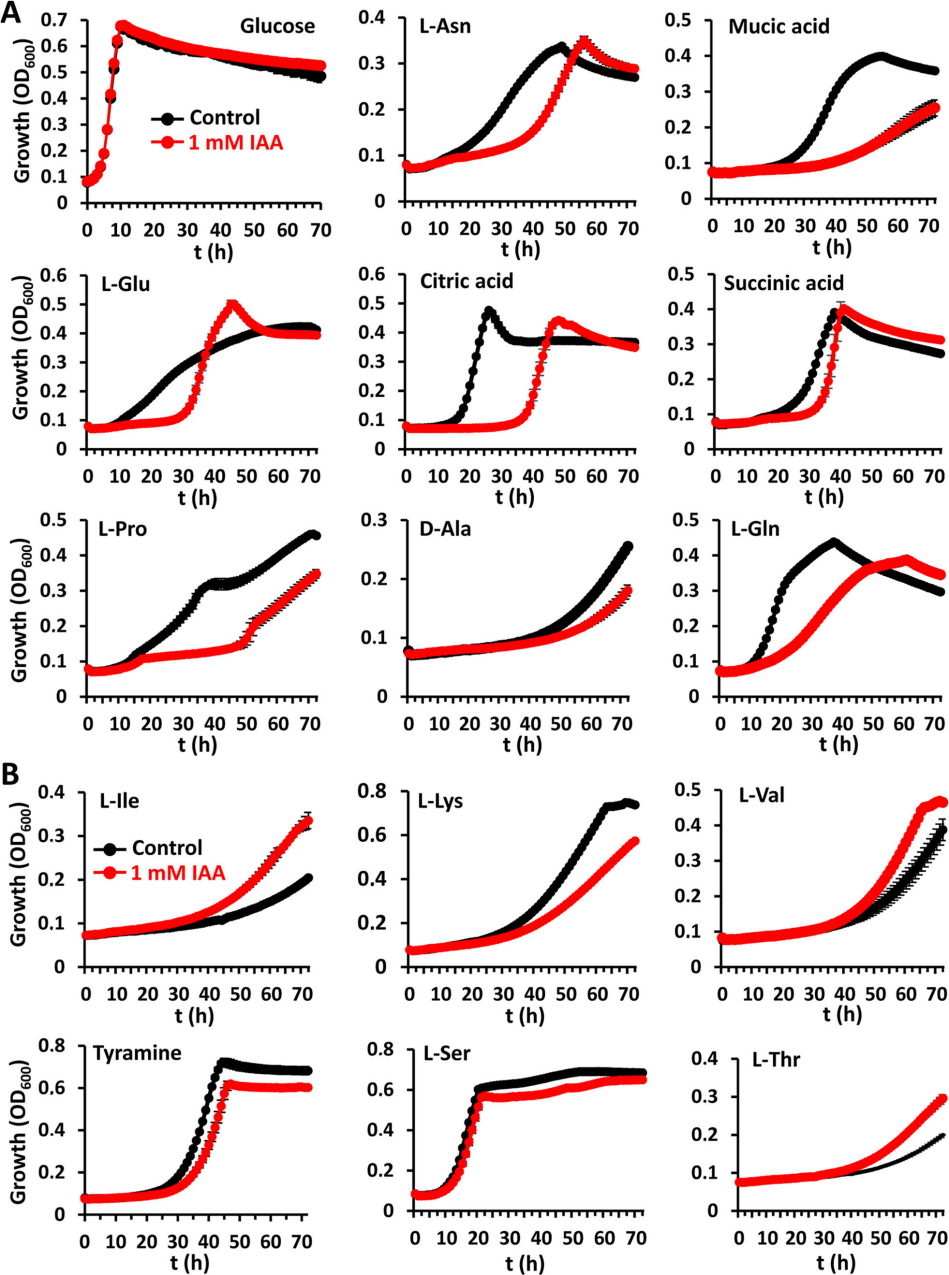
Indole-3-acetic acid affects the metabolism of different nutrients as a sole carbon (**A**) and nitrogen (**B**) sources in *Serratia plymuthica*. In all cases, 10 mM of each carbon and nitrogen source were added to the bacterial cultures. Growth curves in black and red were done in minimal medium supplemented with 15 mM glucose in the absence and presence of 1 mM IAA, respectively. Growth was measured using Bioscreen Microbiological Growth Analyser (Oy Growth Curves Ab Ltd, Helsinki, Finland). Data are the means and standard deviations of five biological replicates. Some standard deviations are minor and are not visible in the corresponding growth curves. The bioassays were repeated three times, and representative results are shown.

Given the large number of DEGs with implications for cellular metabolism and transport of organic and inorganic nutrients (Fig. 2D; Fig. S2; Tables S1 and S2), we investigated the effect of IAA on the metabolic capabilities of A153. First, to identify compounds that may serve as carbon and nitrogen sources, we analyzed growth using commercial Biolog Phenotype MicroArray plates PM1 and PM3B, each containing 95 potential carbon and nitrogen sources, respectively. These metabolites included sugars, sugar phosphates, amino and organic acids, alcohols, purines, pyrimidines, dipeptides, among others. Growth assays showed that A153 is able to use 65% and 77% of the tested metabolites as sole carbon and nitrogen source, respectively (Table S5). Subsequent analyses showed that exposure to 1 mM IAA altered the metabolic capacities for the utilization of 37% of the carbon sources tested (Fig. 3A; Fig. S4; Table S5). Particularly, the metabolism of amino acids, dipeptides, as well as that of aliphatic and aromatic organic acids was mainly affected. In contrast, no significant alteration was observed in the metabolism of other metabolites such as sugars, sugar derivatives, purines, or pyrimidines (Table S5). Alternatively, IAA treatment altered the metabolism of 22% of the nitrogen sources tested, primarily amino acid metabolism. IAA also slightly modulated growth on the pyrimidines thymidine, uracil, and uridine as sole N-sources (Fig. 3B; Fig. S5; Table S5), which contrasted with the above growth experiments when used as sole C-sources. In most cases, IAA decreased the growth rate, with the exception of L-Ala as sole carbon source, and L-Ile, L-Leu, L-Thr, and L-Val as sole nitrogen sources (Fig. 3; Fig. S4 and S5; Table S5).

Since most of the compounds with an altered metabolism in the presence of IAA are present in root exudates (42, 43), we investigated the effect of IAA on the growth kinetics of A153 in maize root exudates, which are rich in amino and organic acids (44). The results revealed that IAA treatment caused a reduction in A153 growth in root exudates (Fig. S6).

### IAA modulates the expression of the AaeXAB efflux pump to confer resistance to toxic aromatic acids and IAA in *Serratia plymuthica*

**Fig 4:**
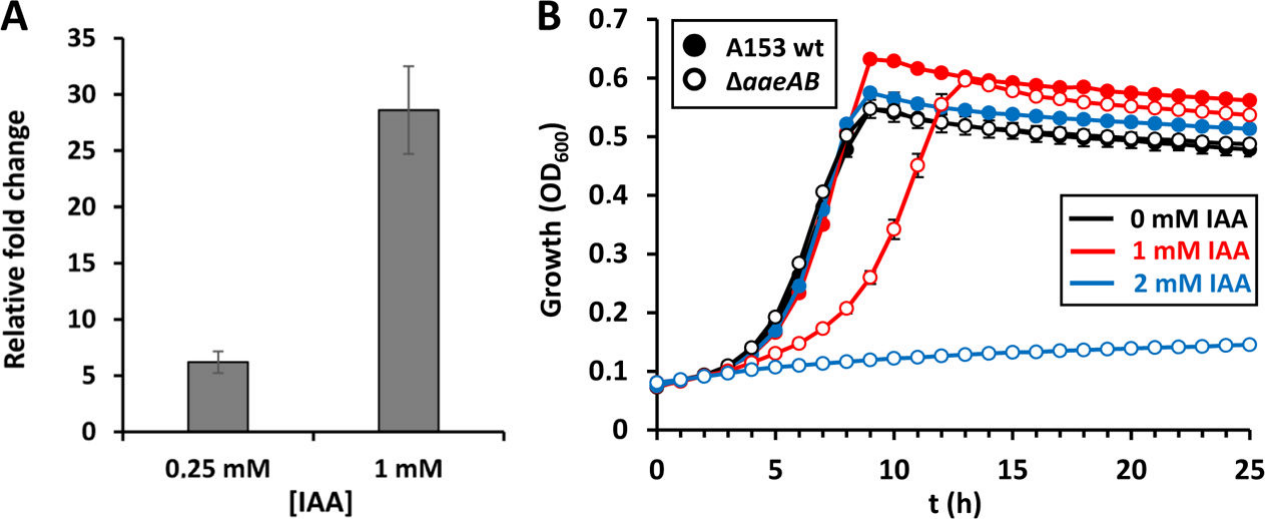
Indole-3-acetic acid (IAA) regulates the expression of the AaeXAB efflux pump to control resistance to high levels of IAA in *Serratia plymuthica*. (**A**) Impact of different IAA concentrations on *aaeX* transcript levels. Shown are the fold-changes of *aaeX* transcript levels measured quantitative RT-PCR under the same conditions used for the RNA-seq analysis, namely treatment with IAA versus cells not exposed to IAA. Data are the means and standard errors of three biological replicates, each conducted in triplicate. (**B**) Growth kinetics of *S. plymuthica* A153 strains with different concentrations of IAA. MIC values are shown in Table S6. Growth experiments were conducted in minimal medium with 15 mM glucose as carbon source at 30°C. Growth was measured using Bioscreen Microbiological Growth Analyser (Oy Growth Curves Ab Ltd, Helsinki, Finland). Data are the means and standard deviations of five biological replicates. Some standard deviations are minor and are not visible in the corresponding growth curves.

Among the genes with the highest levels of induction in response to IAA were those encoding the efflux pump AaeXAB, with induction values ranging from 4.8- to 11.2-fold and 15.3- to 28.2-fold in response to 0.25 mM and 1 mM IAA, respectively (Table 1; Tables S1 and S2). These data were subsequently confirmed by RT-qPCR (Fig. 4A). The AaeXAB efflux pump was identified in *Escherichia coli* as a system mainly involved in 4-hydroxybenzoic acid (4HBA) efflux (45), and AaeXAB proteins of A153 and *E. coli* show identities between 61.5% and 85.1%. To confirm the role of the AaeXAB pump in A153 resistance to 4HBA, we generated a deletion mutant deficient in *aaeAB*. The *aaeAB* mutant grew as the parental strain in the absence of 4HBA, whereas it showed a severely reduced growth in the presence of 50 mM 4HBA, with no growth observed above this concentration [Fig. S7; see Table S6 for minimal inhibitory concentration (MIC) values]. 4HBA is one of the main phenolic acids in root exudates (43, 46) and is an important plant compound that signals to bacteria. 4HBA is sensed by transcriptional regulators to regulate gene expression (1, 47) and plant-derived 4HBA modulated virulence in important phytopathogens (47, 48).

To investigate whether the AaeXAB pump could be acting as a protective mechanism against high IAA concentrations, we evaluated the growth of the *aaeAB* mutant at different IAA concentrations. Whereas no alterations in growth were observed at IAA concentrations up to 5 mM in the wild-type strain (37), the *aaeAB* mutant failed to grow at concentrations above 1 mM IAA (Fig. 4B). We determined the MIC values of IAA for the wild type and the *aaeAB* mutant and found a more than 12-fold reduction in the mutant strain (Table S6), indicating that the AaeXAB pump confers resistance to high levels of IAA. Subsequently, we analyzed the role of IAA on bacterial growth in the presence of high 4HBA concentrations. We observed that IAA affected, in a concentration-dependent manner, the growth of A153 in the presence of high levels of 4HBA with respect to IAA-free control cultures (Fig. S8).

The expression of the *aaeXAB* operon in *E. coli* is controlled by the LTTR AaeR (45). LTTR family members typically consist of a N-terminal DNA-binding domain and a C-terminal ligand-binding domain (LBD). Typically, ligand binding modulates the regulatory activity of LTTRs (49). Previous data in *E. coli* revealed that 4HBA, salicylate, and benzoate induce *aaeXAB* expression (45). However, to our knowledge, no ligands have been identified for AaeR. To address this issue, we overexpressed his-tagged A153 AaeR (AaeR_A153_; 79% identical with the *E. coli* homolog), but the purified protein was unstable under all conditions tested, preventing further analyses. Subsequently, we purified the LBD of AaeR_A153_ as an individual recombinant protein—an approach previously used to identify cognate ligands in other LTTRs (1). AaeR_A153_-LBD was obtained as stable and folded protein, but isothermal titration calorimetry (ITC) studies revealed no binding of 4HBA, salicylate, benzoate, and IAA. Subsequent high-throughput screening of AaeR_A153_-LBD for binding of compounds in arrays PM1, PM2A, PM3B, PM4A, and PM5 also failed to identify its ligands.

### IAA enhances ampicillin resistance in *S. plymuthica*

**Fig 5:**
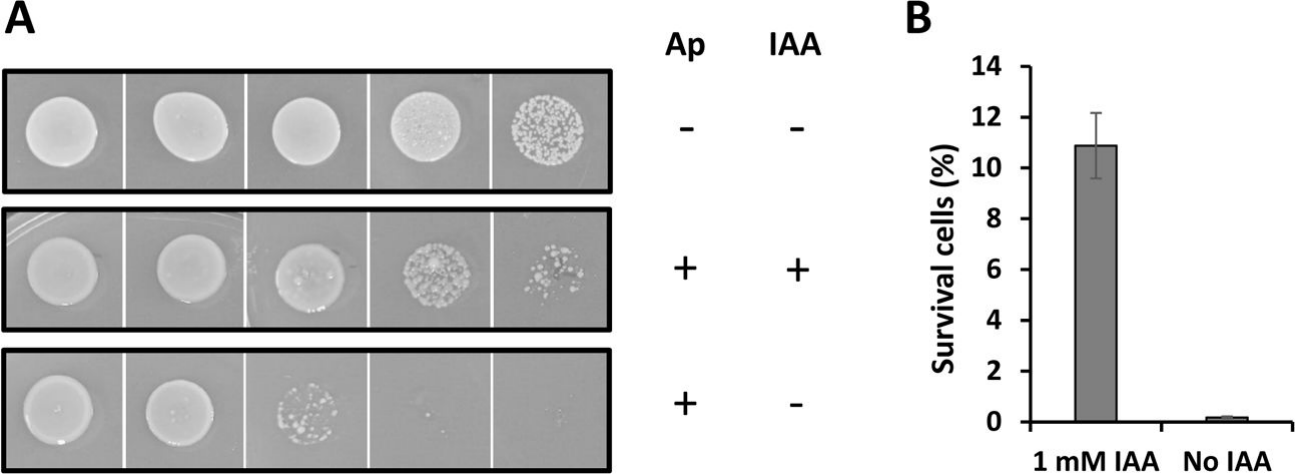
Indole-3-acetic acid (IAA) increases ampicillin resistance in *Serratia plymuthica*. (**A**) Ampicillin (Ap) resistance of A153 in response to 1 mM IAA. Ampicillin was added to the plates at a concentration of 50 µg/mL. (**B**) Quantification of survival cells (%) corresponding to Fig. 5A. Data are the means and standard deviations from three biological replicates.

Several genes that were shown to be involved in antibiotic resistance in other bacterial species were induced in A153 in response to IAA (Table 1; Tables S1 and S2), including those encoding the OmpC porin (50), the multidrug transporter permeases SanA (51) and MdtB (52), the multidrug transporter MacB (53), a multidrug resistance protein (AWY96_RS15045) (54), capsular polysaccharide (CPS) biosynthesis proteins (55), and a β-lactamase (AWY96_RS20405) (56). To investigate the effect of IAA on antibiotic resistance in A153, we determined the MIC values of various antibiotics that operate with different mechanisms of action, namely, ampicillin, chloramphenicol, gentamicin, kanamycin, nalidixic acid, streptomycin, rifampicin, and tetracycline. We found that IAA treatment enhances resistance to gentamicin and kanamycin (Table S6), two aminoglycoside antibiotics that inhibit protein synthesis by binding to the 30S ribosomal subunit (57). Subsequent experiments revealed that IAA significantly increased resistance to ampicillin in minimal medium agar plates by at least an order of magnitude (Fig. 5), which correlates with the increased expression of the β-lactamase encoding gene *AWY96_RS20405*. No significant effects of IAA were observed for the other antibiotics tested (e.g., gentamicin, kanamycin, nalidixic acid, streptomycin, and rifampicin) in minimal medium agar plates. By monitoring growth over time at antibiotic concentrations below the MICs, we found that IAA not only favored growth in the presence of ampicillin, kanamycin, and gentamicin but also with nalidixic acid and streptomycin (Fig. S9). In contrast, exposure to IAA reduced the growth rate in the presence of rifampicin, and no effect was observed with chloramphenicol and tetracycline (Fig. S9).

Because antibiotic tolerance, defined as the ability of bacteria to survive exposure to antibiotics, may not result in changes in MIC values (58), we evaluated the effect of IAA on the survival of A153 cells in exponential phase of growth after treatment with the high concentrations of ampicillin, gentamicin, kanamycin, nalidixic acid, streptomycin, and rifampicin. No differences were observed in the presence and absence of IAA, collectively indicating that this auxin does not induce tolerance to the antibiotics tested.

### IAA treatment reduces c-di-GMP levels altering motility and biofilm formation

**Fig 6:**
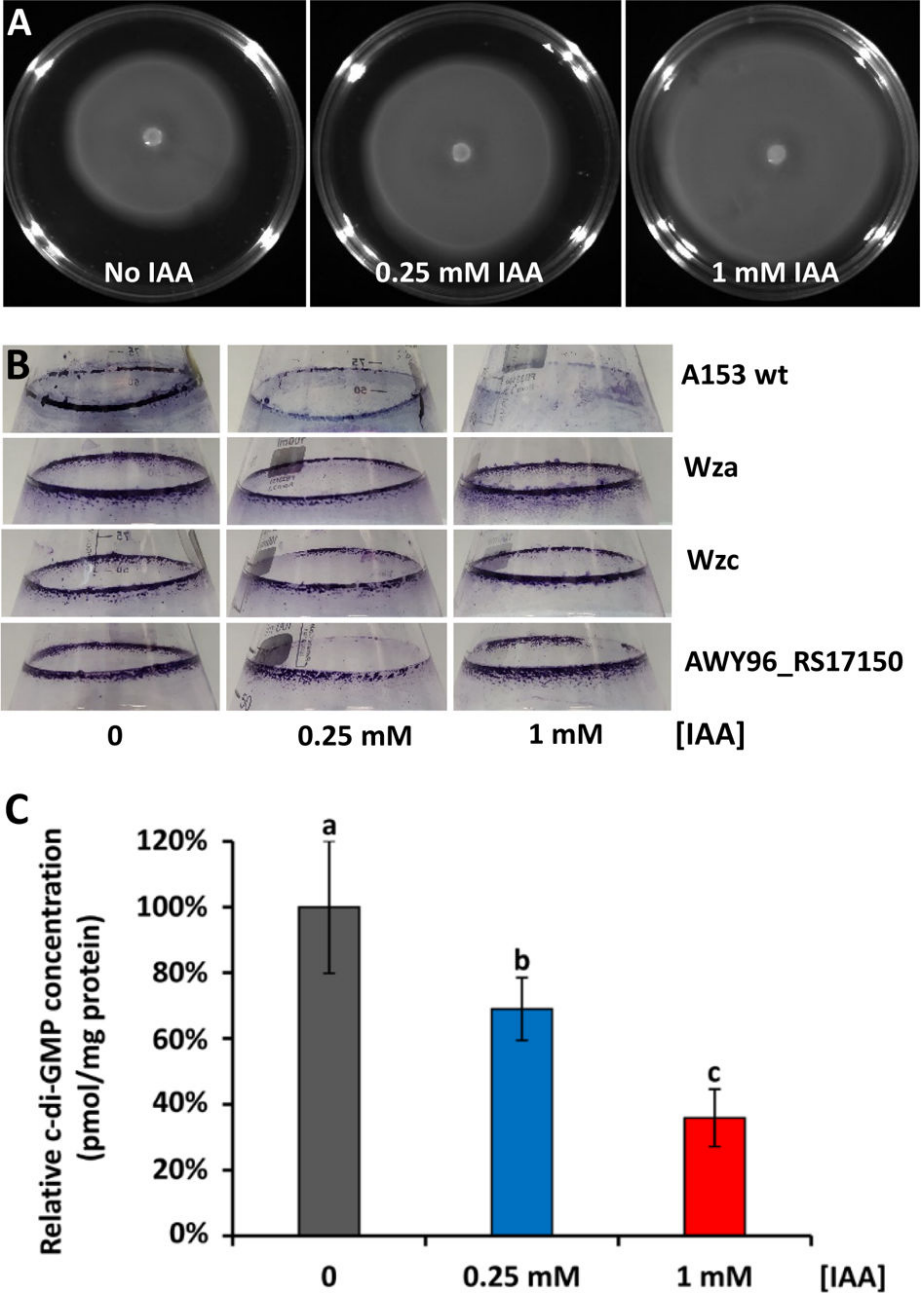
IAA treatment lowers c-di-GMP levels to promote motility and inhibit biofilm formation in *Serratia plymuthica*. (**A**) Swimming motility was examined in minimal medium (0.3% agar, wt/vol) supplemented with 15 mM glucose in the absence and presence of IAA. Pictures were taken after 48 h of growth at 30°C. (**B**) Biofilm formation in borosilicate glass flasks. Bacteria were grown in minimal medium with orbital shaking at 200 rpm. Pictures were taken after 24 h of growth at 30°C. (**C**) Quantification of c-di-GMP levels in A153 by LC-MS/MS. Data are represented as pmol of c-di-GMP per mg total protein and were normalized to the concentration of c-di-GMP in A153 cultures without added IAA. Data are the means and standard deviations of three biological replicates. Bars with the same letter are not significantly different (*P*-value < 0.05; by Student’s *t*-test).

The second messenger c-di-GMP plays an important role in the transition from a motile to a sessile lifestyle in bacteria (59, 60). The identification of several DEGs involved in c-di-GMP turnover and motility (Table 1; Tables S1 and S2) encouraged us to analyze the role of IAA in the modulation of several c-di-GMP-regulated phenotypes. First, we conducted swimming motility assays and observed a significant increase in motility in response to increasing IAA concentrations, causing a ~30% increase in the swimming diameter in the presence of 1 mM IAA (Fig. 6A). Second, we found that IAA inhibited biofilm formation, resulting in a total inhibition at a concentration of 1 mM IAA (Fig. 6B). These motility and biofilm phenotypes are in agreement with a IAA-mediated decrease in c-di-GMP levels. To further investigate this issue, we quantified the global c-di-GMP levels in A153 by liquid chromatography-tandem mass spectrometry (LC-MS/MS). We found that IAA reduced c-di-GMP levels in a concentration-dependent manner, causing a ~31% and ~64% decrease in the presence of 0.25 mM and 1 mM IAA, respectively (Fig. 6C).

IAA exposure caused an upregulation of a ~45 kbp biosynthetic gene cluster (*AWY96_RS17105-AWY96_RS17245*) (Table 1; Table S1) previously found to be involved in the synthesis of the capsular polysaccharide (CPS) in A153 (61). To investigate the role of CPS in the IAA-mediated inhibition of biofilm formation, we phenotypically characterized mutants deficient in CPS synthesis (61), namely in *wza* (encoding an integral outer membrane protein essential for CPS export), *wzc* (encoding a tyrosine autokinase essential for CPS assembly), and *AWY96_RS17150*. The results showed that disruption of CPS production abolishes the IAA inhibitory effects on biofilm formation (Fig. 6B), thus establishing that the observed phenotype is CPS-dependent. The role of CPS in biofilm formation has been previously shown in various bacterial species (62–64) and was associated with blocking surface determinants important for cell adhesion as well as with alterations in the cell surface hydrophobicity and charge (62, 64).

### IAA exposure increases sensitivity to a capsule-dependent phage

**Fig 7:**
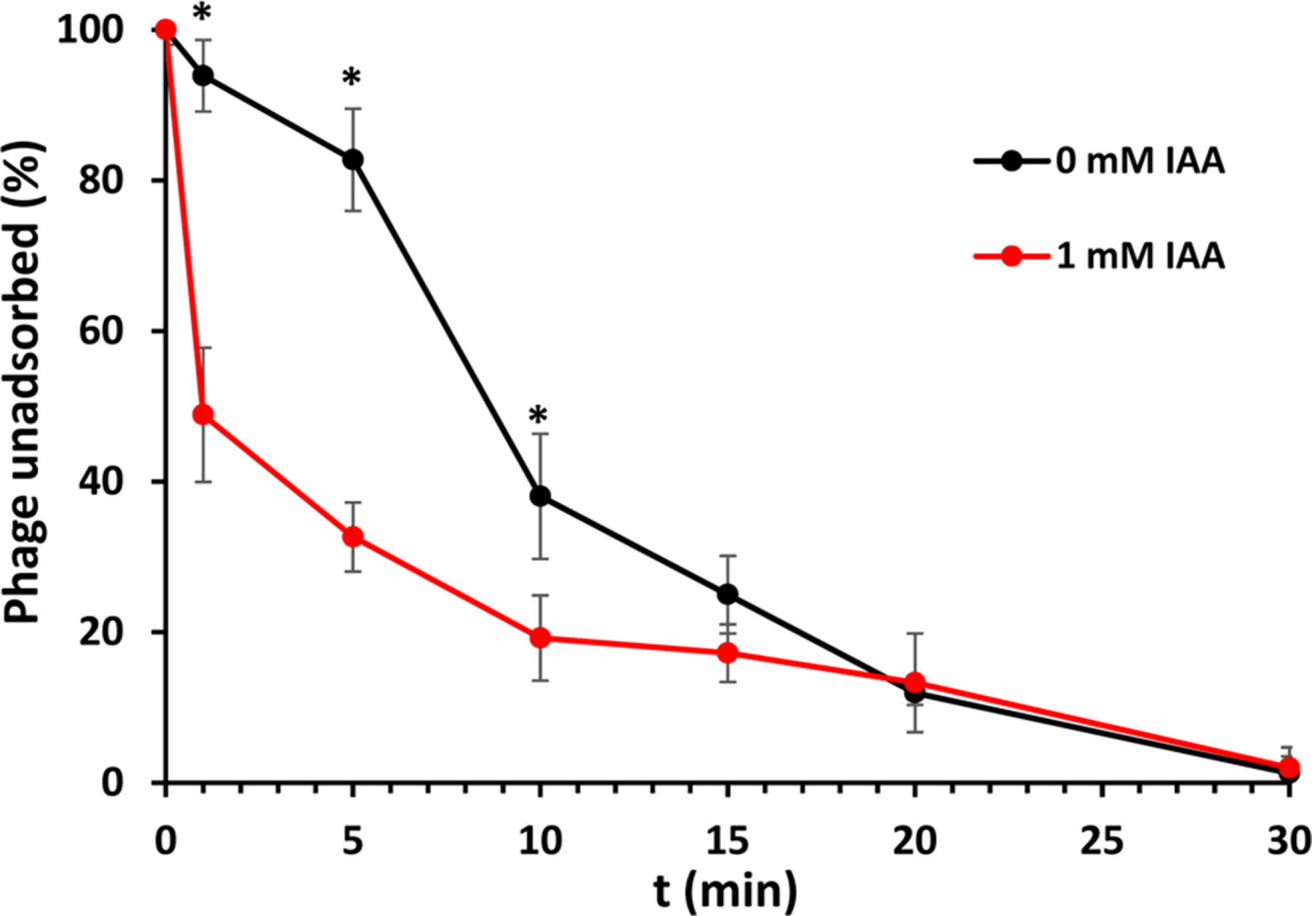
IAA treatment promotes phage attachment to *S. plymuthica* A153. Shown are adsorption assays of ɸMAM1 to A153 in the absence and presence of 1 mM IAA. Data are the means and standard deviations of three assays conducted in triplicate. *P < 0.05, by Student’s *t*-test.

We previously isolated the bacteriophage ɸMAM1 that infects *S. plymuthica* A153 using CPS as receptor (61). To investigate whether the IAA-mediated upregulation of *cps* genes (Table 1; Table S1) led to increased ɸMAM1 attachment onto A153, we performed phage adsorption assays. These assays revealed that IAA treatment caused a more efficient adsorption compared to bacteria not exposed to the auxin (Fig. 7). This phenomenon was especially noticeable during the initial stages of the adsorption process. For example, 68% of phage particles were adsorbed after the first 5 minutes post-mixing on bacteria grown in the presence of 1 mM IAA, whereas only 18% of phage particles became adsorbed within the same time when A153 was not treated with IAA (Fig. 7). Nonetheless, the total number of phages adsorbed on bacteria grown in the presence and absence of IAA was comparable after 20 minutes post-mixing (Fig. 7). Taken together, these results indicate that an enhanced expression of the phage receptor increases phage adsorption rate.

### TrpR_A153_ is an auxin-binding transcriptional regulator whose expression is upregulated by IAA

**Fig 8:**
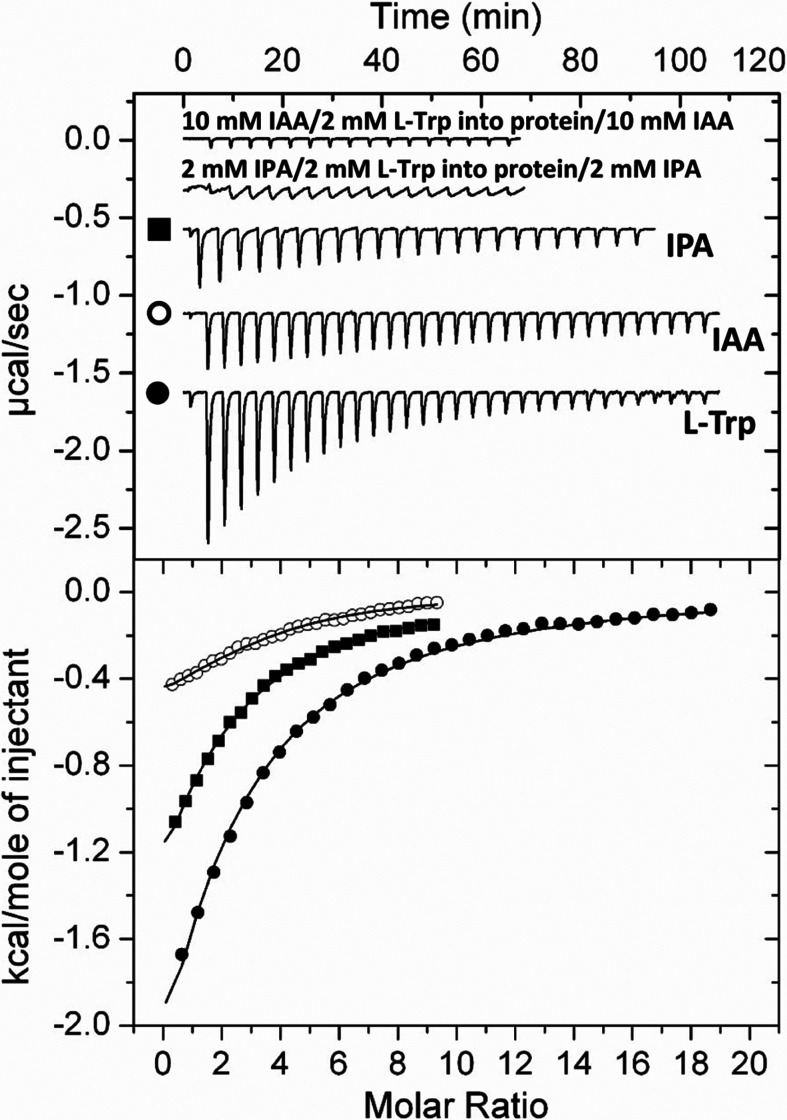
Isothermal titration calorimetry studies of the binding of different ligands to TrpR_A153_ of *Serratia plymuthica* A153. Upper panel: raw data for the titration of 25–50 μM of TrpR_A153_ (AWY96_RS12805) with 9.6- to 12.8-µL aliquots of 1–2 mM ligand solutions. Lower panel: integrated, dilution heat-corrected and concentration-normalized peak areas fitted using the “One binding site” model of the MicroCal version of ORIGIN. Symbols used in the lower panel are defined in the upper panel of this figure. L-Trp, L-tryptophan; IAA, indole-3-acetic acid; IPA, indole-3-pyruvic acid.

Numerous transcriptional regulators encoding genes were up- and downregulated in response to IAA (Fig. 2D; Fig. S2; Tables S1 and S2). Among these genes, we found that *AWY96_RS12805* showed increased expression in the presence of 1 mM IAA, but inter-sample variability caused it not to pass the established *P*adj cutoff. AWY96_RS12805 shares 66% sequence identity with the *E. coli* tryptophan repressor TrpR—a transcriptional regulator previously shown to bind L-Trp and IAA (65) and that primarily regulates aromatic amino acid metabolism and transport (66). Notably, the role of IAA in the expression of TrpR-like regulators has not been previously explored. We conducted RT-qPCR assays and found that *AWY96_RS12805* expression was upregulated 1.9 ± 0.2- and 3.2 ± 0.4-fold in response to 0.25 mM and 1 mM IAA, respectively (Fig. S3). Subsequently, we investigated the ligands that are recognized by AWY96_RS12805. For this purpose, AWY96_RS12805 was purified and submitted to ITC assays that revealed binding of L-Trp and IAA with dissociation constants (*K*_D_) of 91 ± 3 and 326 ± 30 µM, respectively (Fig. 8)—highlighting the potential of transcriptomics to identify IAA-sensing regulatory proteins. As in the case of AdmX (37), AWY96_RS12805 also bound indole-3-pyruvic acid (IPA) with a *K*_D_ of 101 ± 7 µM (Fig. 8). Subsequent differential scanning fluorimetry-based high-throughput screening using a collection of ∼450 compounds that served as carbon, nitrogen, sulfur, or phosphorous sources for bacteria revealed no additional ligands. AWY96_RS12805 was renamed TrpR_A153_.

To investigate whether IAA and IPA compete with L-Trp for binding to TrpR_A153_, we conducted competitive binding assays. We first analyzed the capacity of IAA and IPA to compete for binding to TrpR_A153_ through microcalorimetric titrations. The results revealed that saturating TrpR_A153_ with either IAA or IPA prevented binding of IPA or IAA, respectively, to the regulator (Fig. S10), indicating that both auxins compete for binding to TrpR_A153_. Subsequent experiments revealed an absence of L-Trp binding to TrpR_A153_ in the presence of saturating concentrations of IAA or IPA (Fig. 8). Alternatively, no IAA binding to TrpR_A153_ was noted in the presence of saturating concentration of L-Trp (Fig. S10). Taken together, these results indicate that IPA and IAA compete with L-Trp for their binding to TrpR_A153_. This also implies that changes in L-Trp levels interfere with auxin-mediated signaling. To our knowledge, A153 is the only bacterium in which two auxin-binding transcriptional regulators have been identified and future studies will focus on analyzing the ligands and regulatory cascades of the distinct regulators whose expression is modulated by IAA.

## DISCUSSION

IAA is a multi-faceted signal molecule that exerts a variety of regulatory functions in phylogenetically distant species. Next to its pivotal role in plant growth and development (23–25), it regulates fungal physiology (67), microalgal growth (68), inflammatory and carcinogenic processes in animals and humans (69, 70), and, as shown here, bacterial metabolism and physiology. Among the signals that play a major role in plant environments, IAA is emerging as a key compound, allowing plant-associated microbes to adapt efficiently to their hosts and to establish interactions with other (micro)organisms in plant niches (18–20, 29, 33). However, we are currently only at the beginning of understanding the mechanisms by which IAA regulates bacterial physiology, metabolism and social behavior.

Previous research revealed an effect of exogenous (71–75) and endogenous (29, 39, 73, 76) IAA on the global transcriptomes of different plant-associated bacteria. These studies demonstrated a role of IAA in the modulation of bacterial transcriptomes, including the differential regulation of genes involved in stress responses (29, 74), nitrogen fixation (76), metabolism (39, 72, 73), as well as pathogenesis and virulence factor production (71, 75, 77). These differential and multifaceted regulatory effects, together with the fact that current research on bacterial auxin signaling has focused mainly on plant pathogenic and nitrogen-fixing bacteria, reinforces the need to investigate IAA signaling pathways in different model microorganisms, as well as to advance in the mechanisms by which IAA exerts its activities.

Our results show that IAA causes remarkable changes in the transcriptome of *S. plymuthica*. Among the functional gene categories that show the most important transcriptional changes were those with implications in primary metabolism and energy production. Subsequent high-throughput screening revealed important metabolic changes, primarily in the metabolism of amino and organic acids. In agreement with these results, transcriptomic studies conducted in the phytobacteria *Pseudomonas syringae* (75), *Sinorhizobium meliloti* (76), *Azospirillum brasilense* (73), and *Bradyrhizobium japonicum* (74) showed that genes involved in the metabolism of amino acids, nucleotides, carbohydrates, lipids, or energy metabolism were the most affected by IAA. In this regard, the activities of different enzymes of bacterial central metabolism were altered in response to IAA (72, 78), and IAA was also shown to especially affect energy and amino acid metabolism in antibiotic-resistant *Staphylococcus aureus* (79).

Secondary metabolism represents a metabolic burden for bacteria since it diverts energy, precursors, and cofactors from primary metabolism (80, 81). Andrimid biosynthesis requires the amino acid phenylalanine, glycine, and valine as precursors (82), and we found that IAA alters phenylalanine and valine metabolism—an aspect that may modulate andrimid biosynthesis in response to IAA. In addition, the expression of the andrimid operon was found to be regulated post-transcriptionally (36), and we identified four sRNAs encoded in the andrimid operon that were upregulated by IAA (Table S4). We hypothesize that these sRNAs could act as translational repressors of the andrimid operon, and future research will further assess this hypothesis. Several studies have attributed a role for IAA in the regulation of bacterial secondary metabolism, both activating and repressing antibiotic production (28, 37, 83, 84)—suggesting a role for IAA-mediated signaling in allowing bacteria to thrive in complex and highly competitive niches. Notably, additional phytohormones like jasmonic acid and salicylic acid were also shown to modulate antibiotic production in plant-associated bacteria (83, 85).

Plants have evolved several strategies to control IAA homeostasis (86). Analogously to plants, bacteria have developed various mechanisms to control IAA homeostasis and to counteract possible toxic effects of high IAA concentrations, including auxin catabolism (30, 87), the generation of IAA inactive conjugates (19, 88), and auxin efflux (89). Here, we discovered that the efflux pump AaeXAB, in addition to conferring resistance to 4HBA, also confers resistance to high levels of IAA. To the best of our knowledge, MatE is the only so far known bacterial transporter involved in IAA efflux (89), and future studies will analyze the role of the AaeXAB pump in IAA extrusion. 4HBA is a signal molecule that regulates different features of plant-bacteria interaction, including plant defense against pathogens (90), bacterial phytopathogenicity (47, 91, 92), chemotaxis (91, 93), as well as exopolysaccharide (94) and pigment (92) biosynthesis. We showed that IAA has an important effect on the expression of a 4HBA efflux pump, indicative of a cross-talk between IAA- and 4HBA-controlled regulatory circuits. In addition, we also showed that different auxins (e.g., IPA and IAA) and L-Trp compete for binding to TrpR_A153_, suggesting the existence of a cross-regulation between auxins and amino acids. Together, these findings highlight the complexity of IAA-mediated signaling and illustrate the intricacy of chemical plant-bacteria signaling.

The upregulation of the efflux pump AaeXAB in response to IAA as well as that of genes involved in capsule synthesis and with implications for antibiotic resistance may be indicative of stress. Previous studies have shown that IAA modulates different physiological and metabolic bacterial processes of importance during interaction with plants (19, 20, 33). For example, during plant colonization, bacteria face multiple stresses such as oxidative stress, presence of antimicrobial compounds, and adaptation to specific nutrients (95–98), and IAA has been shown to play a role in adapting to these and other environmental stresses (19, 33). Notably, it has been shown that IAA present in root exudates affects the composition of microbial communities (18) and that plant-associated bacteria exhibit chemotaxis towards IAA (31) and are frequently able to catabolize IAA (30, 99)—highlighting again the biological significance of IAA in mediating plant-bacteria interactions. IAA can be found in the rhizosphere at micromolar concentrations (100, 101), but further studies are needed to determine the IAA concentrations at the microscale in plant niches. By analogy with our data, jasmonic acid, another key phytohormone, was also shown to modulate the physiology of plant-associated bacteria (85).

We showed here that IAA exposure modulates antibiotic susceptibility, which is reminiscent to the observation that IAA increases ampicillin resistance in the biocontrol agent *Pseudomonas putida* by unknown mechanisms (102). In contrast, IAA promoted metabolism in *S. aureus* to reduce antibiotic tolerance (79). Indole was identified as a signal molecule that either increased and decreased antibiotic resistance by modulating the expression of transporter and stress resistance genes, as well as by the formation of persister cells (103, 104). Although further research is needed to identify the mechanisms of IAA-mediated antibiotic tolerance, our findings support an interplay between compound efflux, membrane permeability, enzymatic antibiotic inactivation and a decrease in bacterial metabolism. Our previous research identified with IPA an IAA antagonist that regulated antibiotic synthesis in *S. plymuthica* (38) and the identification of further IAA antagonists may permit the development of new strategies to fight multidrug resistance bacteria.

Since its discovery in the late 1980s, c-di-GMP has emerged as one of the key bacterial second messengers, being involved in the coordination of critical processes such as biofilm formation, motility, cell development, and virulence (59, 60). Although several studies showed that IAA treatment affects biofilm formation (74, 105) and motility (75, 76), the underlying mechanisms remain largely unknown. To the best of our knowledge, we establish here, for the first time, an effect of IAA on c-di-GMP-mediated signaling. IAA decreased c-di-GMP levels which correlated with the increased motility and decreased biofilm-forming capacity observed in this condition. Furthermore, IAA upregulated the expression of the *cps* gene cluster and CPS inhibited biofilm formation in response to IAA. Previous studies established c-di-GMP as a regulator of CPS synthesis (106–108), and we hypothesize that the alteration in *cps* expression is, at least in part, mediated by c-di-GMP signaling in *S. plymuthica*. Future work will be focused on characterizing the c-di-GMP turnover enzymes involved in the IAA-mediated responses.

### Conclusions and future perspectives

Our knowledge of the signaling roles of IAA opens possibilities for different biotechnological and clinical applications (69, 109–111), including (i) the biosynthesis of products of clinical and industrial interest, (ii) the construction of biosensors, (iii) anti-virulence therapies, (iv) GSE248473 identificationof antibiotic adjuvants, and (v) microbiome engineering as a strategy to promote plant and animal health. This study advances our knowledge of the mechanisms by which IAA modulates motility, biofilm formation, bacteriophage sensitivity, and resistance to antimicrobials. Given the role of efflux pumps as virulence factors (112), as well as the re-emerging potential of phage therapy to combat multidrug-resistant infections (113), research derived from this work will pave the way for studies aimed at utilizing auxins as anti-virulence agents to combat multidrug-resistant pathogens.

## MATERIALS AND METHODS

### Strains, bacteriophages, plasmids, oligonucleotides, and culture conditions

Bacteria, phages, and plasmids used in this study are described in Table S7, whereas oligonucleotides are listed in Table S8. *S. plymuthica* strains were grown routinely at 30°C, unless otherwise indicated, in lysogeny broth (LB) or minimal medium [0.1% (wt/vol) (NH_4_)_2_SO_4_, 0.41 mM MgSO_4_, 40 mM K_2_HPO_4_, 14.7 mM KH_2_PO_4_, pH 6.9–7.1] with 15 mM glucose as carbon source, unless otherwise indicated. *E. coli* strains were grown at 37°C in LB. *E. coli* DH5α was used as a host for gene cloning. Media for propagation of *E. coli* β2163 were supplemented with 300 µM 2,6-diaminopimelic acid. When appropriate, antibiotics were used at the following final concentrations (in µg mL^−1^): ampicillin, 100; kanamycin, 50; streptomycin, 50—unless otherwise stated. Sucrose was added to a final concentration of 10% (wt/vol) when required to select derivatives that had undergone a second crossover event during marker-exchange mutagenesis.

### *In vitro* nucleic acid techniques

Plasmid DNA was isolated using the NZY-Miniprep kit (NZY-Tech). For DNA digestion, alkaline phosphatase, and ligation reactions, manufacturers’ instructions were followed (New England Biolabs and Roche). DNA fragments were recovered from agarose gels using the Qiagen gel extraction kit. PCRs were purified using the Qiagen PCR Clean-up kit. PCR fragments were verified by DNA sequencing that was carried out at the Institute of Parasitology and Biomedicine Lopez-Neyra (CSIC; Granada, Spain). Transformations and electroporations were performed using standard protocols (114). Phusion high-fidelity DNA polymerase (Thermo Fisher Scientific) was used for the amplification of PCR fragments.

### Marker exchange mutagenesis

A deletion mutant defective in *aaeAB* was constructed by homologous recombination using a derivative plasmid of the suicide vector pKNG101. The resulting plasmid pMAMV403 (Table S7) was transferred to *S. plymuthica* A153 by biparental conjugation using *E. coli* β2163. All plasmids and the resultant ∆*aaeAB* mutant strain were confirmed by PCR and sequencing.

### Phenotypic assays

Antagonistic activities against *Bacillus subtilis* were conducted as described previously (36). Swimming assays were performed on minimal medium-Difco agar (0.3% [wt/vol]) plates at 30°C. Biofilm assays were carried out in 100-mL flasks with 20 mL bacterial cultures grown in minimal medium under orbital shaking (200 rpm) at 30°C. After 24 h, bacterial cultures were removed, flasks rinsed with water, and biofilms stained with crystal violet (0.5%, wt/vol) for 15 min at room temperature. β-Galactosidase assays were carried out as previously described (115).

### Collection of root exudates

Maize seeds were sterilized and germinated as described previously (96) and root exudates were collected from 16 germinated seeds as previously indicated (44). Maize root exudates were freeze-dried and stored at −80°C until use.

### Growth experiments and antibiotic susceptibility assays

A153 was grown overnight in minimal medium containing 15 mM glucose. Overnight cultures were washed twice and then diluted to an OD_600_ of 0.02 in minimal medium containing either: (i) 15 mM glucose supplemented with different concentrations of antibiotics, (ii) 15 mM glucose supplemented with different concentrations of 4HBA and/or IAA, or (iii) each of the compounds present in the Biolog (Hayward, CA, USA) compound arrays PM1 and PM3B as sole carbon source and nitrogen sources, respectively. Growth in root exudates was done in 50 mM phosphate buffer in the presence of 10× and 100× maize root exudates, concentrations that correspond to 2.5 and 25 g/L freeze-dried exudates, respectively. Differences in the growth of A153 in the absence and presence of 1 mM IAA were considered when measuring alterations in growth rate, lag phase, and/or when the maximum OD_600_ reached in growth experiments varied by at least 10%. In all cases, 200 µL of the cultures were transferred into microwell plates, and growth at 30°C was followed on a Bioscreen microbiological growth analyzer (Oy Growth Curves Ab Ltd., Helsinki, Finland).

Minimal inhibitory concentration (MIC) assays were performed in minimal medium supplemented with 15 mM glucose in the presence and absence of 1 mM IAA using a twofold serial dilution test (116). The MIC was established as the lowest concentration of a compound that prevented growth in liquid cultures after 48 h at 30°C. Growth was followed on a Bioscreen microbiological growth analyzer (Oy Growth Curves Ab Ltd., Helsinki, Finland). Alternatively, the effect of IAA on antibiotic resistance was also assessed in solid media. Briefly, serial dilutions of overnight cultures grown in minimal medium in the presence and absence of 1 mM IAA were spot-plated onto minimal medium agar supplemented with antibiotics or both 1 mM IAA and antibiotics (50–150 μg/mL ampicillin, 1–12 μg/mL gentamicin, 3–25 μg/mL kanamycin, 1–5 μg/mL nalidixic acid, 3–25 μg/mL streptomycin, and 5–20 μg/mL rifampicin). Serial dilution plates were allowed to grow overnight at 30°C.

Antibiotic tolerance was assessed in minimal medium supplemented with 15 mM glucose. Briefly, overnight cultures in minimal medium were used to inoculate fresh medium with and without 1 mM IAA to reach an OD_600_ of 0.1. Cells were then cultured at 30°C until an OD_600_ of 0.4, at which time the cultures were challenged with different antibiotics (100–400 μg/mL ampicillin, 10 µg/mL gentamicin, 50 µg/mL kanamycin, 10–20 μg/mL nalidixic acid, 50 µg/mL streptomycin, and 10–40 μg/mL rifampicin) for a period from 1 to 3 h. Survival was determined by serial dilution plating comparing the colony counts before and after antibiotic treatment.

### Phage adsorption assays

Phage adsorption assays were conducted as described previously (61), with minor modifications. Briefly, an overnight bacterial culture of A153 was adjusted to an OD_600_ of 0.1 in minimal medium in the presence and absence of 1 mM IAA. After overnight growth, 5 mL cultures were then infected with ɸMAM1 at a multiplicity of infection of 0.01, mixed briefly, and placed on a tube roller at 25°C. A bacterium-free negative control was created by adding the same amount of phage to 5 mL of minimal medium. One-hundred-microliter samples were removed at different times and added to 900 µL of phage buffer [10 mM Tris-HCl, 10 mM MgSO_4_, 0.01% (wt/vol) gelatin, pH 7.4] containing 30 µL of chloroform. The components were mixed for 5 s and centrifuged at 13,000 × *g* for 1 min. The number of unadsorbed phage particles was determined by titrating serial dilutions of the supernatants on LB agar (0.35%, wt/vol, agar) lawns. Phage adsorption was expressed as a percentage of the number of plaque-forming units (PFU) mL^−1^ in the bacterium-free negative control.

### c-di-GMP quantification by liquid chromatography-tandem mass spectrometry

A153 cultures grown overnight in minimal medium were used to inoculate 100-mL flasks containing 20 mL of fresh medium with and without 0.25 and 1 mM IAA to an OD_600_ of 0.1. After 16 h of growth at 30°C, 10 mL samples were harvested by centrifugation at 2,500 × *g* for 20 min at 4°C. The resulting cell pellets were resuspended in 300 µL of an ice cold extraction solvent mixture (acetonitrile/methanol/water, 2/2/1, vol/vol/vol), incubated on ice for 15 min, and heated at 95°C for 10 min. Cells were then centrifuged at 20,800 × *g* for 10 min at 4°C, and the supernatants were collected. This extraction was repeated twice, each time using 200 µL of the extraction solvent. The supernatants from the three extractions were combined and incubated at −20°C overnight for protein precipitation. After overnight incubation, the samples were centrifuged at 20,800 × *g* for 10 min at 4°C. The final extracts were dried in a speed-vac system and analyzed by LC-MS/MS at the Biolog Life Science Institute GmbH & Co. KG (Bremen, Germany), following a previously described method (117). Samples were compared to a c-di-GMP standard curve and data were normalized against the quantity of total protein content determined using the Micro BCA Protein Assay Kit (Thermo Scientific; Ref. 23235).

### RNA extraction, cDNA synthesis, and quantitative real-time PCR analyses

Total RNA was extracted using TRI Reagent (Invitrogen) followed by Turbo DNase treatment (Ambion) and RNA clean-up with RNeasy Mini Kit (Qiagen) according to manufacturers’ instructions. RNA degradation and contamination were assessed by electrophoresis on 2% (wt/vol) agarose gels. The synthesis of cDNA was performed using random hexamers (GE Healthcare) and SuperScript II reverse transcriptase (Invitrogen) in a 25 µL reaction volume with 1 µg of total RNA and incubation at 42°C for 2 h. RT-qPCRs were performed as described previously (36) using primers described in Table S8. RT-qPCR amplifications were performed using the iQ SYBR Green supermix (Bio-Rad) in an MyiQ2 Two-Color Real-Time PCR Detection System (Bio-Rad) associated with iQ5 optical system software (version 2.1.97.1001). To confirm the absence of contaminating genomic DNA, control PCRs were carried out using no RT cDNA samples as templates. Melting curve analyses were conducted to ensure the amplification of a single product. The relative gene expression was calculated using the critical threshold (ΔΔCt) method (118) using the *gyrB* gene as reference for data normalization. RT-qPCR validation assays were done using the same samples used for RNA sequencing.

### RNA-seq and data analysis

RNA sequencing was done at the GENYO Research Center (Granada, Spain). Prior to preparation of the RNA library, ribosomal RNAs were removed from the samples using the RiboZero Magnetic Kit (Epicentre; Ref. MRZGN126) following the manufacturers’ instructions. Subsequently, samples were processed with the TruSeq Stranded Total RNA Library Prep Kit (Illumina) following the provided sample preparation guide. The final library (adapter and index included) was validated using the DNA-specific chip Agilent DNA 1000. The final products were fragments between 265 and 300 bp. RNA-seq libraries were sequenced on the Illumina NextSeq 500 sequencer. Initial quality control checks were carried out using FastQC software on the raw sequences (http://www.bioinformatics.babraham.ac.uk/projects/fastqc/). Read mapping and quantification were carried out with the EDGE-pro program (119). Sequences were aligned with the reference genome *S. plymuthica* A153 (35). Batch effect was corrected with the ARSyNSeq function of the NOISeq package (120). Raw counts were normalized using the trimmed mean of *M*-values (TMM) method (121). Differential expression analyses were performed with the DESeq2 package (122). A gene was considered differentially expressed when a false discovery rate (FDR) < 0.05 was observed. The Rockhopper program (123) was used to analyze the sRNAs.

### Protein overexpression and purification

*E. coli* BL21(DE3) harboring plasmids pET29b-TrpR_A153_, pET29b-AaeR and pET28b-AaeR-LBD were grown in 2-L Erlenmeyer flasks containing 500 mL LB medium supplemented with kanamycin. Cultures were grown under continuous stirring (200 rpm) at 30°C. In all cases, at an OD_600_ of 0.5, protein expression was induced by the addition of 0.25 mM isopropyl-β-d-thiogalactopyranoside (IPTG), and growth was continued overnight at 18°C, prior to cell harvest by centrifugation at 20,000 × *g* for 20 min. Proteins were purified by metal affinity chromatography. Briefly, the cell pellets of TrpR_A153_ and AaeR-LBD were resuspended in buffer A [20 mM Tris, 300 mM NaCl, 0.1 mM EDTA, 10% (vol/vol) glycerol, 2 mM β-mercaptoethanol, 10 mM imidazole; pH 8.0], whereas AaeR was resuspended in buffer B [20 mM Tris, 500 mM NaCl, 5% (vol/vol) glycerol, 2 mM β-mercaptoethanol, 10 mM imidazole; pH 7.5] containing cOmplete^TM^ protease inhibitor (Roche) and Benzonase (Merck). Cells were then broken by French press treatment at a gauge pressure of 62.5 lb/in^2^. After centrifugation at 10,000 × *g* for 1 h, the supernatant was loaded onto a 5 mL HisTrap column (Amersham Bioscience) equilibrated with buffers A or B. Proteins were eluted by a gradient of 40 to 500 mM imidazole in the same buffers.

### Isothermal titration calorimetry

Measurements were made using a VP-ITC microcalorimeter (MicroCal, Inc., Northampton, MA) at 25°C. TrpR_A153_ was dialyzed into 5 mM Tris, 5 mM pipes, 5 mM MES, 150 mM NaCl, 10% (vol/vol) glycerol, pH 8.0. Alternatively, AaeR-LBD was dialyzed into 20 mM HEPES, 150 mM NaCl, 2 mM DTT, 5% (vol/vol) glycerol, pH 7.5. Proteins at 50–100 µM were placed into the sample cell and titrated with 6.4–12.8 µL aliquots of 1–5 mM ligand solutions made up in the corresponding dialysis buffers. In all cases, the mean enthalpies measured from the injection of the ligand in the buffer were subtracted from raw titration data before data analysis with the ORIGIN software (MicroCal).

### Differential scanning fluorimetry

Assays were performed on a MyiQ2 real-time PCR instrument (Bio-Rad), as previously described (37). Ligands from the PM1, PM2A, PM3B, PM4A, and PM5 compound arrays (Biolog, Hayward, CA, USA) were dissolved in 50 µL of Milli-Q water, which, according to the manufacturer, corresponds to a concentration of 10–20 mM.

## Data Availability

RNA-seq data were deposited in the Gene Expression Omnibus (GEO) repository under the accession number GSE248473.

## SUPPLEMENTAL MATERIAL

Table S1 msystems.00165-24-s0001.xlsx Genes modulated by the addition of 1 mM IAA.

Table S2 msystems.00165-24-s0002.xlsx Genes modulated by the addition of 0.25 mM IAA.

Table S3 msystems.00165-24-s0003.xlsx Common genes modulated by the addition of 0.25 and 1 mM IAA.

Table S4 msystems.00165-24-s0004.xlsx sRNAs identified in transcriptional analysis in response to 0.25 and 1 mM IAA.

Table S5 msystems.00165-24-s0005.xlsx Growth of *Serratia plymuthica* A153 in minimal medium with different nutrients as sole carbon and nitrogen sources.

Supplemental material msystems.00165-24-s0006.docx Tables S6 to S8 and Figures S1 to S10.

ASM does not own the copyrights to Supplemental Material that may be linked to, or accessed through, an article. The authors have granted ASM a non-exclusive, world-wide license to publish the Supplemental Material files. Please contact the corresponding author directly for reuse.
