# Supplementary material for: Auxin-mediated regulation of susceptibility to toxic metabolites, c-di-GMP levels, and phage infection in the rhizobacterium Serratia plymuthica
Source: mSystems. 2024 Jun 5;9(7):e00165-24. doi: 10.1128/msystems.00165-24 (PMC11264596; doi:10.1128/msystems.00165-24)
Supplement: Supplemental material — Tables S6 to S8 and Figures S1 to S10. [file msystems.00165-24-s0006.docx]

**Supplementary material to:**

**Auxin-mediated regulation of susceptibility to toxic metabolites, c-di-GMP levels and phage infection in the rhizobacterium *Serratia plymuthica***

**Miriam Rico-Jiménez^a^, Zulema Udaondo^a,b^, Tino Krell^a^, Miguel A. Matilla^a^**^#^

^a^Department of Biotechnology and Environmental Protection, Estación Experimental del Zaidín, Consejo Superior de Investigaciones Científicas, Prof. Albareda 1, Granada 18008, Spain.

^b^Department of Biomedical Informatics, University of Arkansas for Medical Sciences, Little Rock, AR 72205, USA.

^#^Address correspondence to:

Miguel A. Matilla, Estación Experimental del Zaidín (CSIC), Granada, Spain, Phone: + 34 958 526506, Fax: + 34 958 135740, e-mail: [miguel.matilla@eez.csic.es](mailto:miguel.matilla@eez.csic.es)

**ORCID numbers Míriam Rico-Jiménez: 0000-0002-0126-1478**

**Zulema Udaondo: 0000-0003-3445-6842**

**Tino Krell: 0000-0002-9040-3166**

**Miguel A. Matilla:** **0000-0002-8468-9604**

**Running title:** Auxin signaling in *Serratia plymuthica*

**Supplementary Tables**

**Supplementary Table S6. Minimal inhibitory concentration (MIC) values for several antibiotics, indole-3-acetic acid (IAA) and 4-hydroxybenzoic acid (4HBA) in *Serratia plymuthica* A153 strains.**

| **Kanamycin**  **(μg/mL)** | | **Gentamicin**  **(μg/mL)** | | **Nalidixic acid**  **(μg/mL)** | | **Ampicillin**  **(μg/mL)** | | **Streptomycin**  **(μg/mL)** | | **Rifampicin**  **(μg/mL)** | | **Tetracycline**  **(μg/mL)** | | **Chloramphenicol**  **(μg/mL)** | | **IAA**  **(mM)** | | **4HBA**  **(mM)** | | |
| --- | --- | --- | --- | --- | --- | --- | --- | --- | --- | --- | --- | --- | --- | --- | --- | --- | --- | --- | --- | --- |
| - | | IAA^a^ | - | IAA^a^ | - | IAA^a^ | - | IAA^a^ | - | IAA^a^ | - | IAA^a^ | - | IAA^a^ | - | IAA^a^ | A153 | ∆*aaeAB* | A153 | ∆*aaeAB* |
| 50 | | 100 | 12.5 | 25 | >40 | >40 | >400 | >400 | 100 | 100 | 40 | 40 | 25 | 25 | >60 | >60 | 25 | 2 | 150 | 75 |

^a^Cultures were supplemented with 1 mM IAA.

**Supplementary Table S7. Bacteria, phage and plasmids used in this study.**

| **Bacterial strains** | **Genotype or relevant characteristic^a^** | **Reference or source** |
| --- | --- | --- |
| *Escherichia coli* DH5α | *supE44 lacU169*(*Ø80lacZΔ M15*) *hsdR17* (r_K_^−^m_K_^−^) *recA1* *endA1* *gyrA96 thi-1* *relA1* | (1) |
| *E. coli* CC118λpir | *araD*, Δ(*ara*, *leu*), Δ*lacZ*74, *pho*A20, *galK*, *thi-1*, *rspE*, *rpoB*, *argE*, *recA1*, λ*pir* | (2) |
| *E. coli* BL21(DE3) | F^–^ *ompT* *gal* *dcm* *lon* *hsdS_B_*(*r_B_*^–^*m_B_*^–^) λ(DE3 [*lacI* *lacUV5*-*T7p07* *ind1* *sam7* *nin5*]) [*malB*^+^]_K-12_(λ^S^) | (3) |
| *E. coli* β2163 | F^-^ RP4-2-Tc::Mu ∆*dapA*::(*erm-pir*); Km^R^, Em^R^ | (4) |
| *Serratia plymuthica* A153 LacA | Δ*lacZ* (1470 bp Δ). Wild type strain | (5) |
| *Serratia plymuthica* VN2 | A153 transposon mutant *admK*::Tn-KRCPN1*lacZ* (transcriptional fusion); Km^R^ | (6) |
| *S. plymuthica* A153 Δ*aaeAB*-Km | Δ*lacZ* Δ*aaeAB*::Km (1410 bp Δ); Km^R^ | This study |
| *S. plymuthica* A153 AWY96_RS17150 | Δ*lacZ* transposon mutant *AWY96_RS17150*::Tn-KRCPN1; capsular polysaccharide defective; Km^R^ | (7) |
| *S. plymuthica* A153 Wzc | Δ*lacZ* transposon mutant *wzc*::Tn-KRCPN1; *wzc* = *AWY96_RS17125*; capsular polysaccharide defective; Km^R^ | (7) |
| *S. plymuthica* A153 Wza | Δ*lacZ* transposon mutant *wza*::Tn-KRCPN1; *wza* = *AWY96_RS17115*; capsular polysaccharide defective; Km^R^ | (7) |
| *Bacillus subtilis* JH646 | *pheA1 trpC2* | J.A. Hoch |
| **Phage** | | |
| ɸMAM1 | *Serratia* infecting phage | (7) |
| **Plasmids** | | |
| pKNG101 | Sm^R^; *oriR6K mob sacBR* | (8) |
| pUC18Not | Ap^R^; identical to pUC18 but with two NotI sites flanking pUC18 polylinker | (2) |
| p34S-Km3 | Ap^R^, Km^R^; *Km3* antibiotic cassette | (9) |
| pET28b(+) | Km^R^; Protein expression plasmid | Novogene |
| pET29b(+) | Km^R^; Protein expression plasmid | Novogene |
| pMAMV397 | Ap^R^; 1.4-kb PCR product containing a 1410 bp deletion of *aaeA* and *aaeB* genes of A153 inserted into the EcoRI/HindIII sites of pUC18Not | This study |
| pMAMV400 | Ap^R^, Km^R^; 0.95 kb BamHI fragment containing *km3* cassette of p34S-Km3 was inserted into the BamHI site of pMAMV397 | This study |
| pMAMV403 | Sm^R^, Km^R^; 1.5-kb NotI fragment of pMAMV400 was cloned at the same site in pKNG101 | This study |
| pET29b-AaeR^b^ | Km^R^; pET29b(+) derivative containing a DNA fragment encoding AaeR (AWY96_RS07110) of A153. C*-*terminal His_6_*-*tag. | This study |
| pET28b-AaeR-LBD | Km^R^; pET28b(+) derivative containing a DNA fragment encoding the ligand binding domain (LBD) of AaeR (AWY96_RS07110) of A153. N*-*terminal His_6_*-*tag. | This study |
| pET29b-TrpR_A153_^b^ | Km^R^; pET29b(+) derivative containing a DNA fragment encoding TrpR_A153_ (AWY96_RS12805) of A153. C*-*terminal His_6_*-*tag. | This study |

### *^a^*Em, erythromycin; Ap, ampicillin; Km, kanamycin; Sm, streptomycin.

### ^b^Constructed by GenScript.

**Supplementary Table S8. Oligonucleotides used in this study.**

| **Name** | **Sequence (5´- 3')** | **Description** | **Source** |
| --- | --- | --- | --- |
| AaeB-EcoRI-F | aaagaattcgagcgatgatcaggttcagcg | Forward primer to clone a region of *AWY96_RS07095*  (*aaeB*) for *aaeAB* deletion in A153 | This study |
| AaeB-BamHI-R | aaaggatccTCAGCGCAGTGGAAGAAGATG | Reverse primer to clone a region of *AWY96_RS07095* (*aaeB*) for *aaeAB* deletion in A153 | This study |
| AaeA-BamHI-F2 | CATCTTCTTCCACTGCGCTGAGGATCCTTTgctgtggacgttcaggttgg | Forward primer to clone a region of *AWY96_RS07100* (*aaeA*) for *aaeAB* deletion in A153 | This study |
| AaeA-HindIII-R | taataagcttCTGGTATCGCTGGCGCTG | Reverse primer to clone a region of *AWY96_RS07100* (*aaeA*) for *aaeAB* deletion in A153 | This study |
| AaeR-LBD-NdeI-F | taatCATATGagcgaggtgcacgaacagc | Forward primer to clone the ligand binding domain encoding region of AaeR (AWY96_RS07110) into pET28b(+) | This study |
| AaeR-LBD-HindIII-R | taataagcttTCACTGATAGACTTCCGCCACGCG | Reverse primer to clone the ligand binding domain encoding region of AaeR (AWY96_RS07110) into pET28b(+) | This study |
| GyrB-qPCR-F | gacgacatcggtgtggaagtg | Forward primer for qRT-PCR. *gyrB* (AWY96_RS09875) gene | This study |
| GyrB-qPCR-R | GGTCATCGCGGTACGGAAAC | Reverse primer for qRT-PCR. *gyrB* (AWY96_RS09875) gene | This study |
| TrpR-qPCR-F | gatgcaactgttgctgacgcc | Forward primer for qRT-PCR. *trpR*_A153_ (*AWY96_RS12805*) gene | This study |
| TrpR-qPCR-R | CGAACCGCGAGTGATGGTG | Reverse primer for qRT-PCR. *trpR*_A153_ (*AWY96_RS12805*) gene | This study |
| AaeX-qPCR-F | atggtcatcttcggactgtcg | Forward primer for qRT-PCR. *aaeX* (*AWY96_RS07105*) gene | This study |
| AaeX-qPCR-R | CAGACAGCAATACAGCGCAG | Reverse primer for qRT-PCR. *aaeX* (AWY96_RS07105) gene | This study |
| RS02175-qPCR-F | gataccgccgataccatcctgc | Forward primer for qRT-PCR. *lrhA/hexS* (*AWY96_RS02175*) gene | This study |
| RS02175-qPCR-R | CCGTCGTAATCGCCAGATCG | Reverse primer for qRT-PCR. *lrhA/hexS* (*AWY96_RS02175*) gene | This study |
| RS21110-qPCR-F | gcgacctcgattgaacgtagc | Forward primer for qRT-PCR. *tonB* (*AWY96_RS21110*) gene | This study |
| RS21110-qPCR-R | CGAGTCGTAATCGCTCTGGTAAC | Reverse primer for qRT-PCR. *tonB* (*AWY96_RS21110*) gene | This study |
| RS17145-qPCR-F | TCTGTCGCAATATGGAAGGAGG | Forward primer for qRT-PCR. *AWY96_RS17145* gene | This study |
| RS17145-qPCR-R | GGAATGACCAAATTCTAACCGATAGC | Reverse primer for qRT-PCR. *AWY96_RS17145* gene | This study |
| RS19815-qPCR-F | tgagactggcggagcaatatg | Forward primer for qRT-PCR. *AWY96_RS19815* gene | This study |
| RS19815-qPCR-R | CAGCTCGTCAGTGAGGATCG | Reverse primer for qRT-PCR. *AWY96_RS19815* gene | This study |
| RS01355-qPCR-F | tccctgccgaggaggaattatc | Forward primer for qRT-PCR. *AWY96_RS01355* gene | This study |
| RS01355-qPCR-R | CACCAACGGCTTGACGTAGG | Reverse primer for qRT-PCR. *AWY96_RS01355* gene | This study |
| RS01440-qPCR-F | ccgtctggtcgacatcgttg | Forward primer for qRT-PCR. *AWY96_RS01440* gene | This study |
| RS01440-qPCR-F | CGCCACCAACACCAGTTCG | Reverse primer for qRT-PCR. *AWY96_RS01440* gene | This study |
| AlsS -qPCR-F | ggataccgtcagcatgttccg | Forward primer for qRT-PCR. *alsS* (*AWY96_RS02860*) gene | This study |
| AlsS-qPCR-R | CCATCGGCAAGCTGATGAACG | Reverse primer for qRT-PCR *alsS* (*AWY96_RS02860*) gene | This study |
| RS17155-qPCR-R | CTTCAGGTGGTTCATAATGGCG | Forward primer for qRT-PCR. *AWY96_RS17155* gene | This study |
| RS17155-qPCR-R | AACTTCGTCATTGCATCCAGC | Reverse primer for qRT-PCR. *AWY96_RS17155* gene | This study |
| RS21015-qPCR-F | GCTATGACGGCGGTCAGTG | Forward primer for qRT-PCR. *AWY96_RS21015* gene | This study |
| RS21015-qPCR-R | CTTCCGCCACGATCTCAATCTC | Reverse primer for qRT-PCR. *AWY96_RS21015* gene | This study |
| RS01340-qPCR-F | CCATCCAGAAGCAGAACCAGAG | Forward primer for qRT-PCR. *AWY96_RS01340* gene | This study |
| RS01340-qPCR-R | CTCCAATTTAGTCAGTCGCAAAGTC | Reverse primer for qRT-PCR. *AWY96_RS01340* gene | This study |
| RS16810-qPCR-F | GTGTCAACGTCATTGAAAGCCTG | Forward primer for qRT-PCR. *AWY96_RS16810* gene | This study |
| RS16810-qPCR-R | GGATGCGGGTAGAGACCAATTC | Reverse primer for qRT-PCR. *AWY96_RS16810* gene | This study |
| RS12635-qPCR-F | ggcgcaggaaatcgatgcag | Forward primer for qRT-PCR. *AWY96_RS12635* gene | This study |
| RS12635-qPCR-R | CATCTCAAGATAAGCCCGACAGC | Reverse primer for qRT-PCR. *AWY96_RS12635* gene | This study |

**SUPPLEMENTARY FIGURES**

**
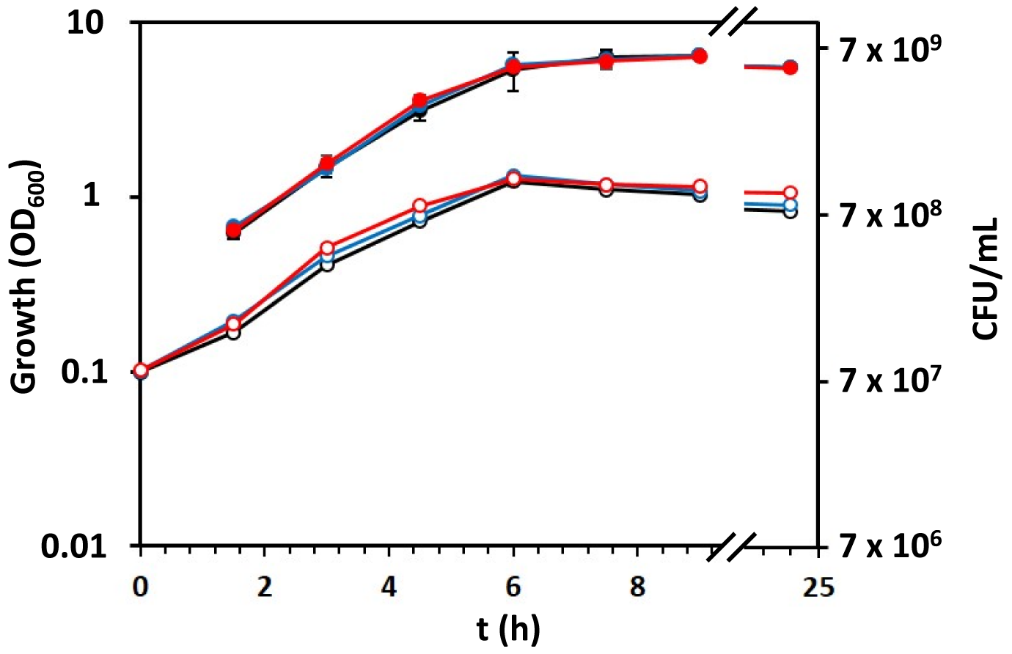
**

**Supplementary Figure S1. Effect of different IAA concentrations on *Serratia plymuthica* A153 growth.** Growth experiments were done under the same conditions in which the bacterial cells were grown for the transcriptomic assays, namely in 100 mL flasks containing 20 mL of minimal medium supplemented with 15 mM glucose as carbon source, under orbital shaking (200 rpm) at 25 ºC. Open symbols represent bacterial growth (OD_600_), whereas the filled symbols represent colony forming units per mL (CFU/mL) throughout growth. Data are the means and standard errors of three biological replicates. Some standard deviations are minor and are not visible in the corresponding growth curves.

**
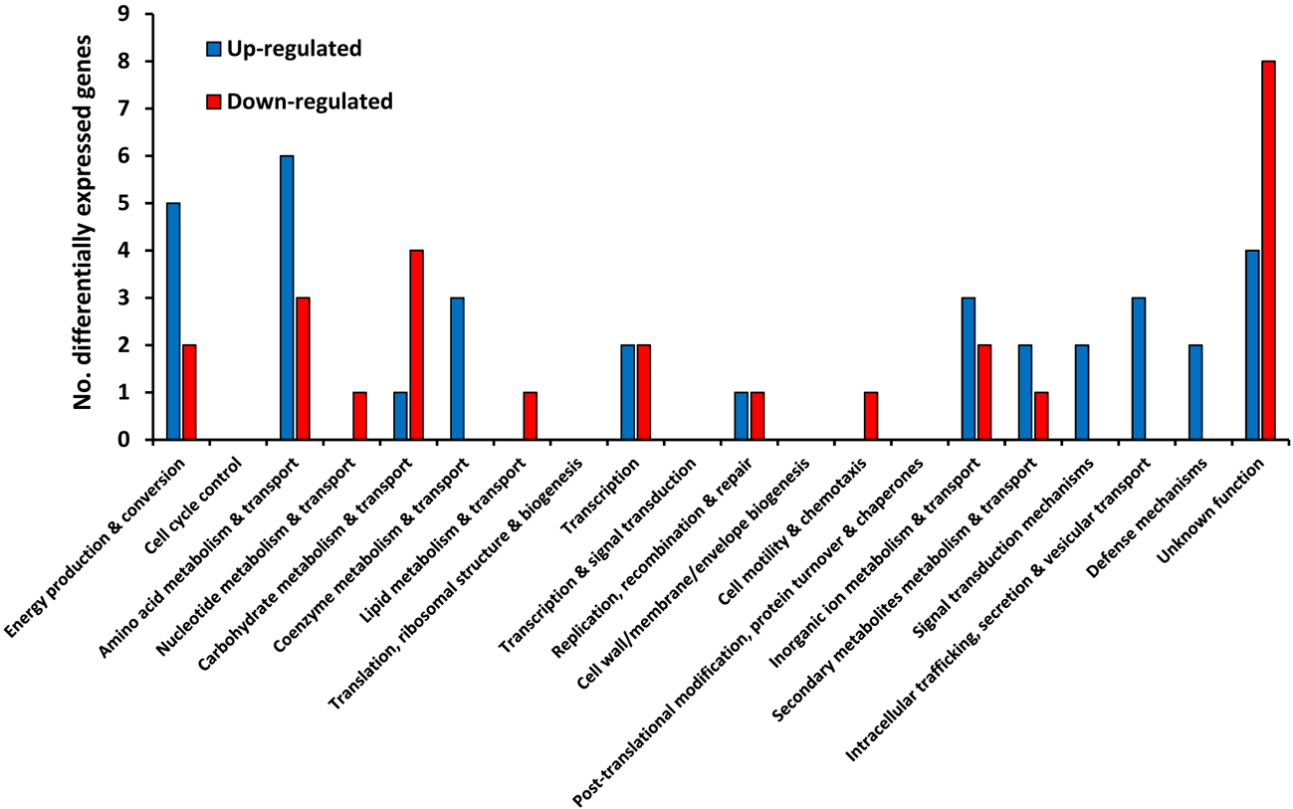
**

**Supplementary Figure S2. Functional classification of the differentially regulated genes in response to 0.25 mM indole-3-acetic acid.** Functional categories were classified according to clusters of orthologous genes (COGs). For clarity, some functional categories according to COGs were combined, as specified in supplementary Table S2.

**
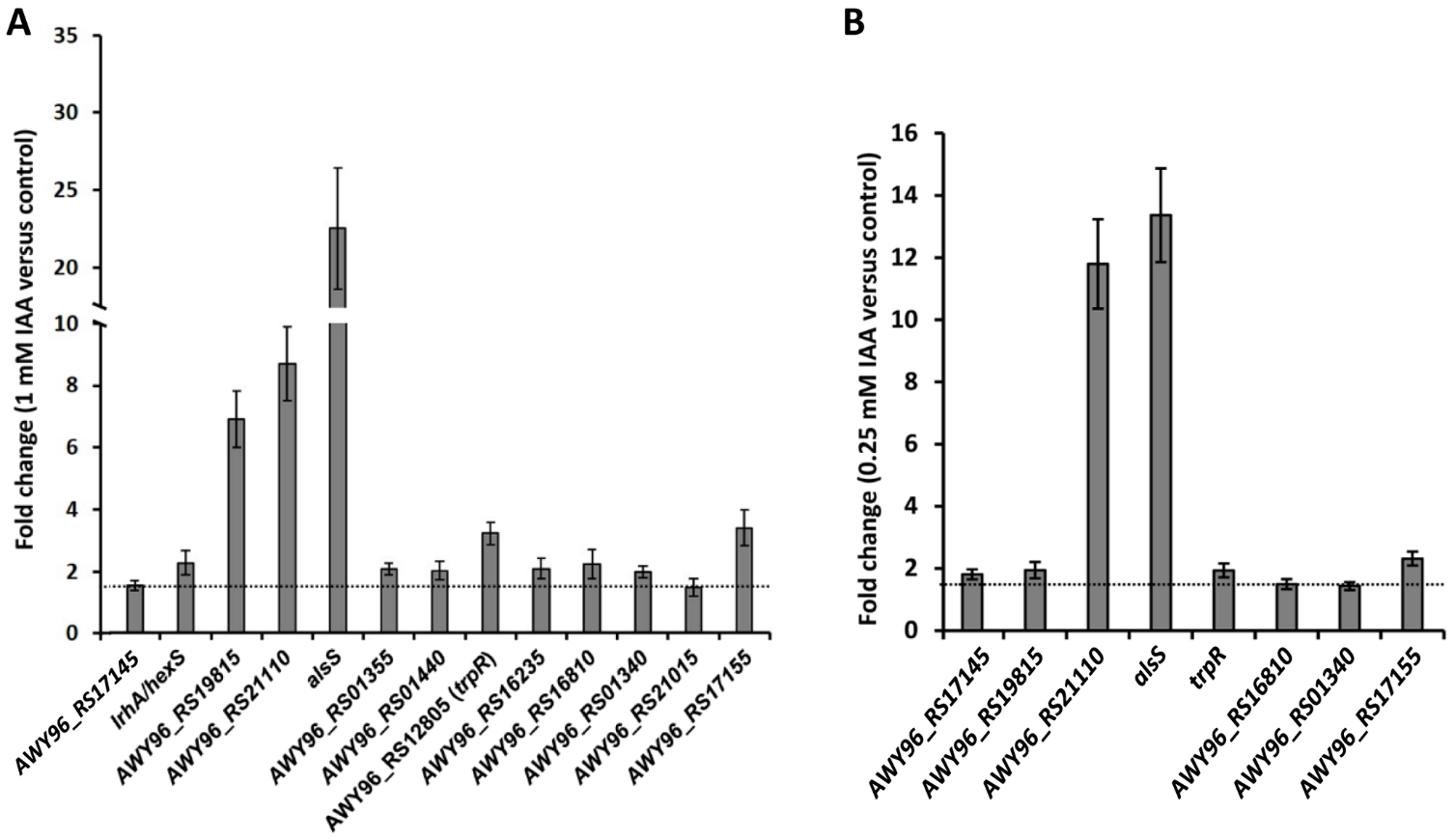
**

**Figure S3. Impact of indole-3-acetic acid on the transcript levels of differentially expressed genes identified in the RNA-seq analysis.** Shown are the fold change of mRNA levels of selected differentially expressed genes in response to 1 mM (**A**) and 0.25 mM (**B**) IAA compared to control measured by quantitative RT-PCR under the same conditions as used for the RNA-seq analysis. Data are the means and standard errors of three biological replicates, each conducted in triplicate. The line represents a fold change of 1.5. LrhA/HexS, transcriptional regulator; AWY96_RS17145, glycosyltransferase family protein; AWY96_RS19815, EAL domain-containing protein; AWY96_RS21110, TonB-dependent receptor; AWY96_RS02860, acetolactate synthase AlsS; AWY96_RS01355, GH3 promoter family protein; AWY96_RS01440, LysR-type transcriptional regulator; TrpR, trp operon repressor; AWY96_RS16235, TetR family transcriptional regulator; AWY96_RS16810, LuxR-like transcriptional regulator; AWY96_RS01340, MmyB-like transcriptional regulator; AWY96_RS21015, LysR-type transcriptional regulator; AWY96_RS17155, glycosyltransferase family protein.


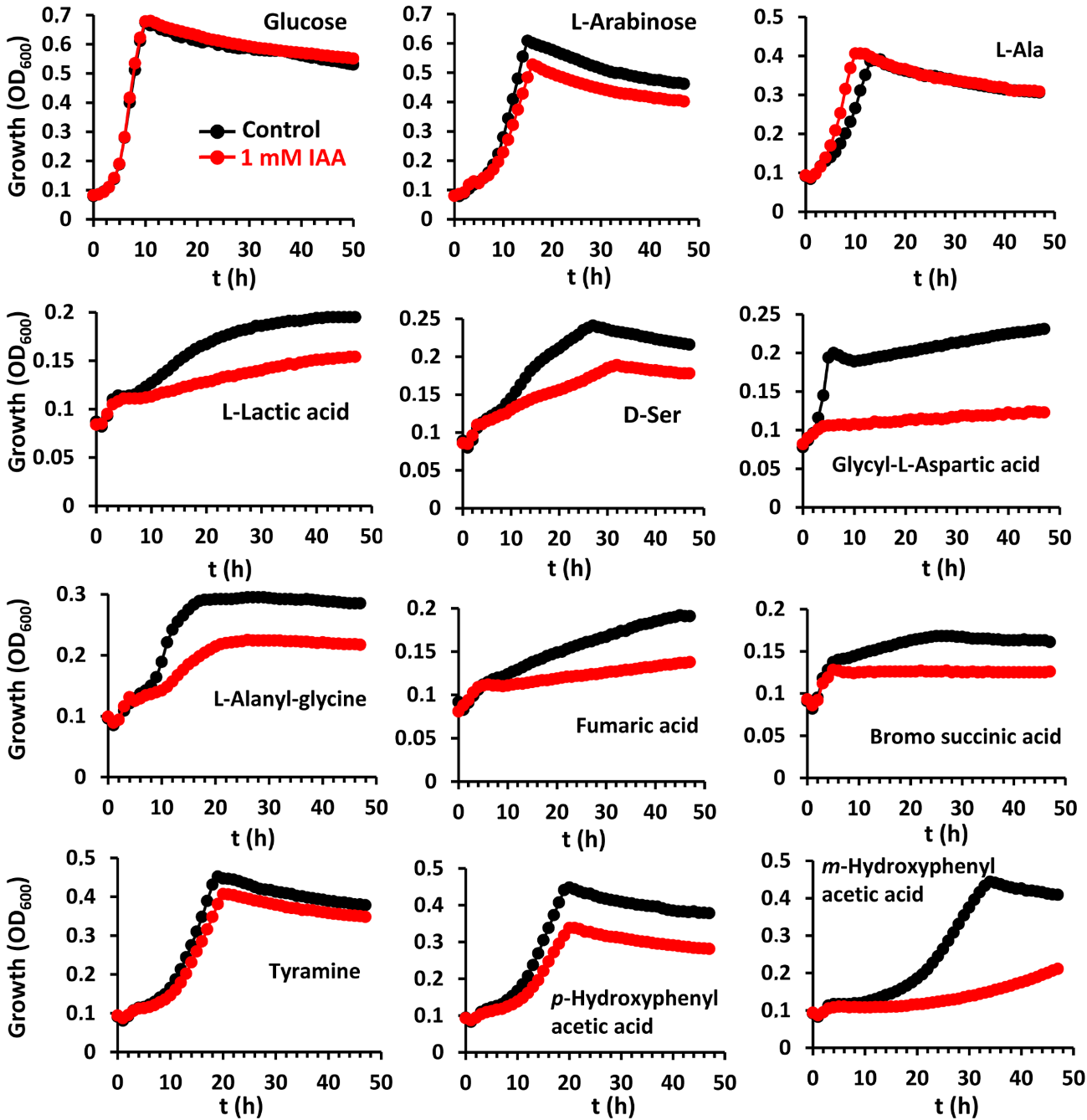


**Supplementary Figure S4. Indole-3-acetic acid affects the metabolism of different nutrients as sole carbon source in *Serratia plymuthica* A153.** In all cases, growth curves in black and red were done in minimal medium in the absence and presence of 1 mM IAA, respectively. Growth was measured using Bioscreen Microbiological Growth Analyser (Oy Growth Curves Ab Ltd, Helsinki, Finland). Data are the means and standard deviations of five biological replicates. Some standard deviations are minor and are not visible in the corresponding growth curves. The bioassays were repeated three times, and representative results are shown.


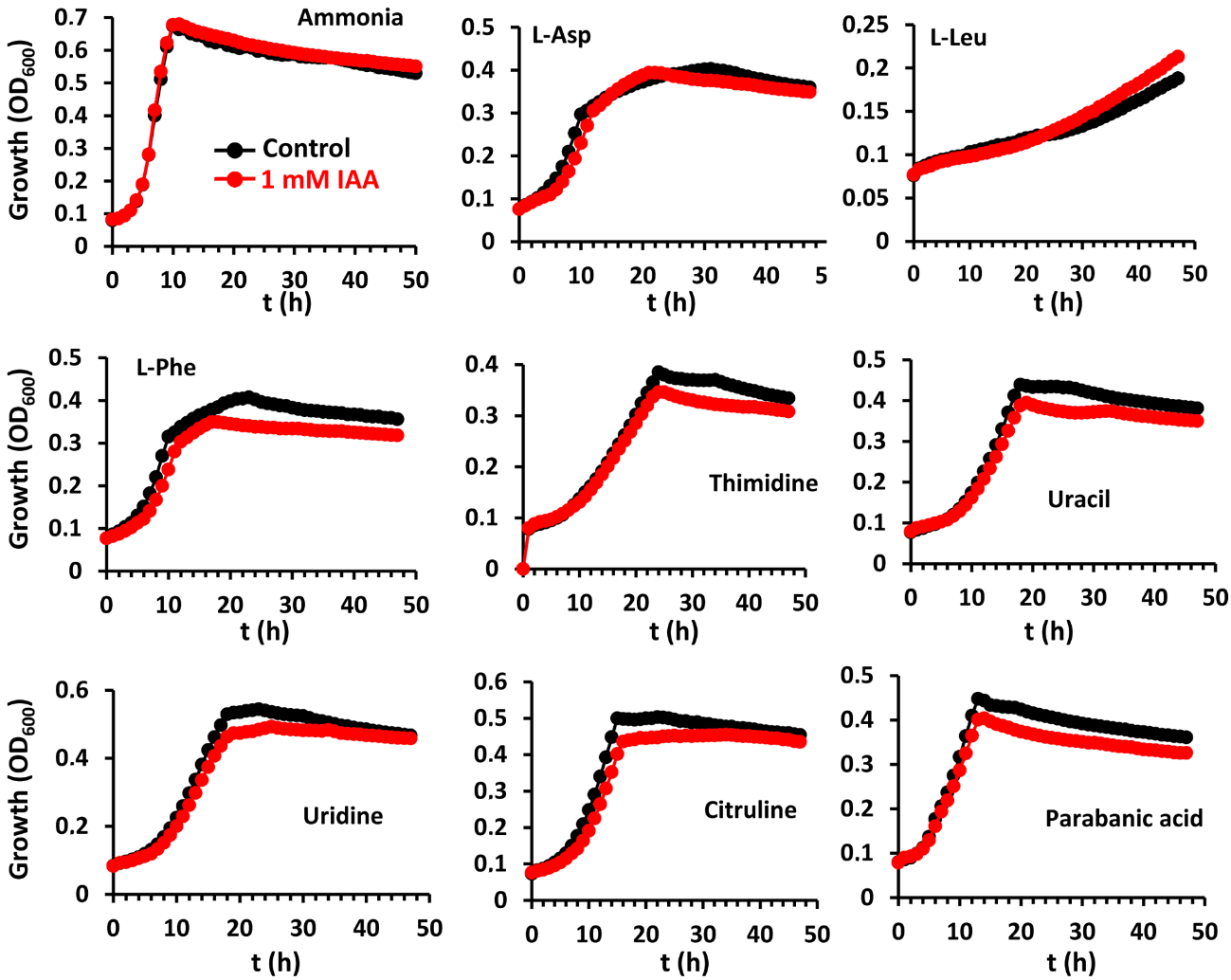


**Supplementary Figure S5. Effect of indole-3-acetic acid on the metabolism of different nutrients as sole nitrogen source in *Serratia plymuthica* A153.** In all cases, growth curves in black and red were done in minimal medium in the absence and presence of 1 mM IAA, respectively. Growth was measured using Bioscreen Microbiological Growth Analyser (Oy Growth Curves Ab Ltd, Helsinki, Finland). Data are the means and standard deviations of five biological replicates. Some standard deviations are minor and are not visible in the corresponding growth curves. The bioassays were repeated three times, and representative results are shown.


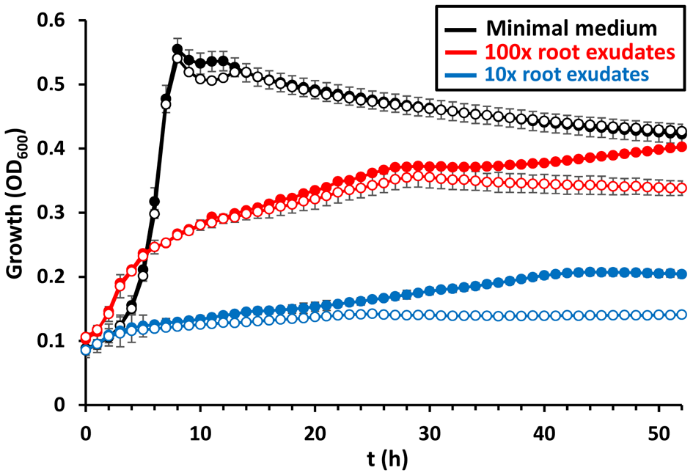


**Supplementary Figure S6. Effect of indole-3-acetic acid on the growth of *Serratia plymuthica* A153 in maize root exudates as sole nutrient source.** Shown is the growth of A153 in different concentrations of maize root exudates in the absence (filled symbols) and presence (empty symbols) of 1 mM of indole-3-acetic acid. Minimal medium with 15 mM glucose as carbon source was used as internal control. Growth was measured using Bioscreen Microbiological Growth Analyser (Oy Growth Curves Ab Ltd, Helsinki, Finland). Data are the means and standard deviations from five biological replicates. Some standard deviations are minor and are not visible in the corresponding growth curves.


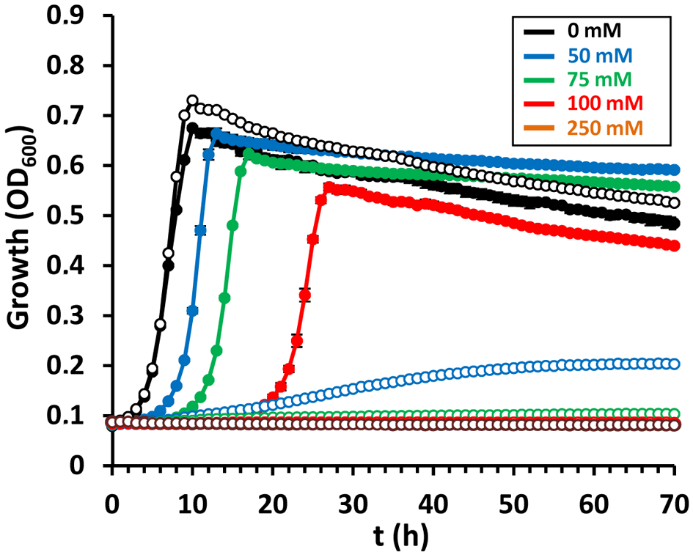


**Supplementary Figure S7. Effect of different concentrations of 4-hydroxybenzoic acid (4HBA) on the growth kinetics of *Serratia plymuthica* A153 strains.** Growth of *S. plymuthica* A153 (filled symbols) and its ∆*aaeAB* mutant (empty symbols) in minimal medium with 15 mM glucose as carbon source at 30 ºC in the absence of presence of different concentrations of 4HBA. Data are the means and standard deviations of five biological replicates. Some standard deviations are minor and are not visible in the corresponding growth curves. The assays were repeated three times and a representative assay is shown. Growth was measured using Bioscreen Microbiological Growth Analyser (Oy Growth Curves Ab Ltd, Helsinki, Finland).


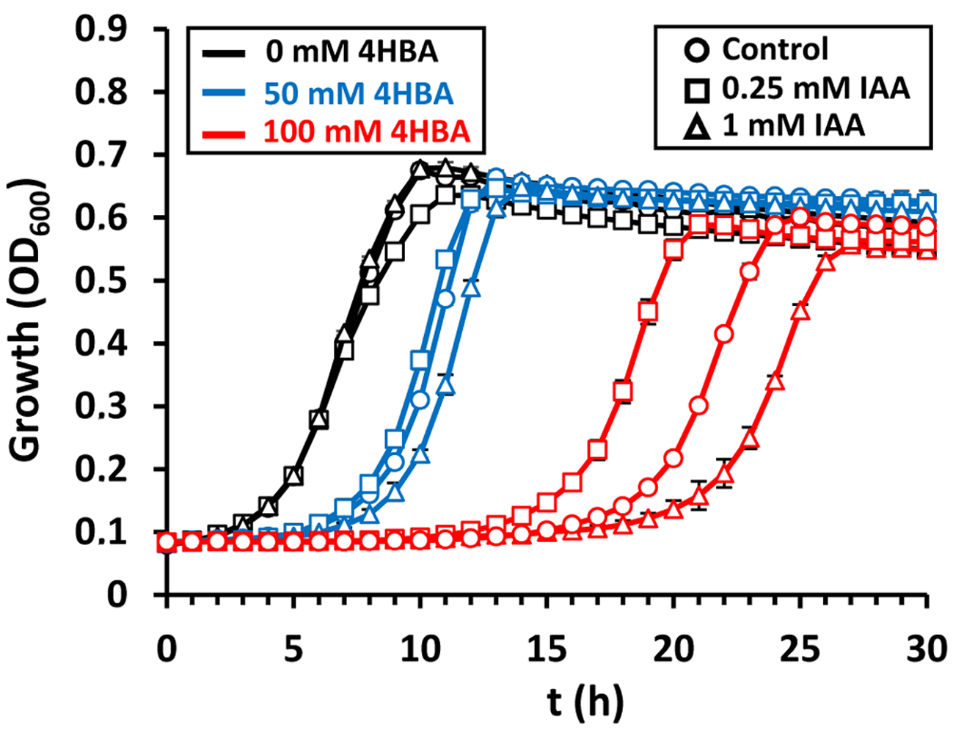


**Supplementary Figure S8. Effect of IAA on the growth of *Serratia plymuthica* A153 in high concentrations of 4-hydroxybenzoic acid (4HBA)**. Shown is the growth kinetics of *S. plymuthica* A153 in minimal medium supplemented with 15 mM glucose and different 4HBA concentrations in the absence and presence of IAA. Growth experiments were done in minimal medium with 15 mM glucose as carbon source at 30 ºC. Growth was measured using Bioscreen Microbiological Growth Analyser (Oy Growth Curves Ab Ltd, Helsinki, Finland). Data are the means and standard deviations of five biological replicates. Some standard deviations are minor and are not visible in the corresponding growth curves. The assays were repeated three times and a representative assay is shown.


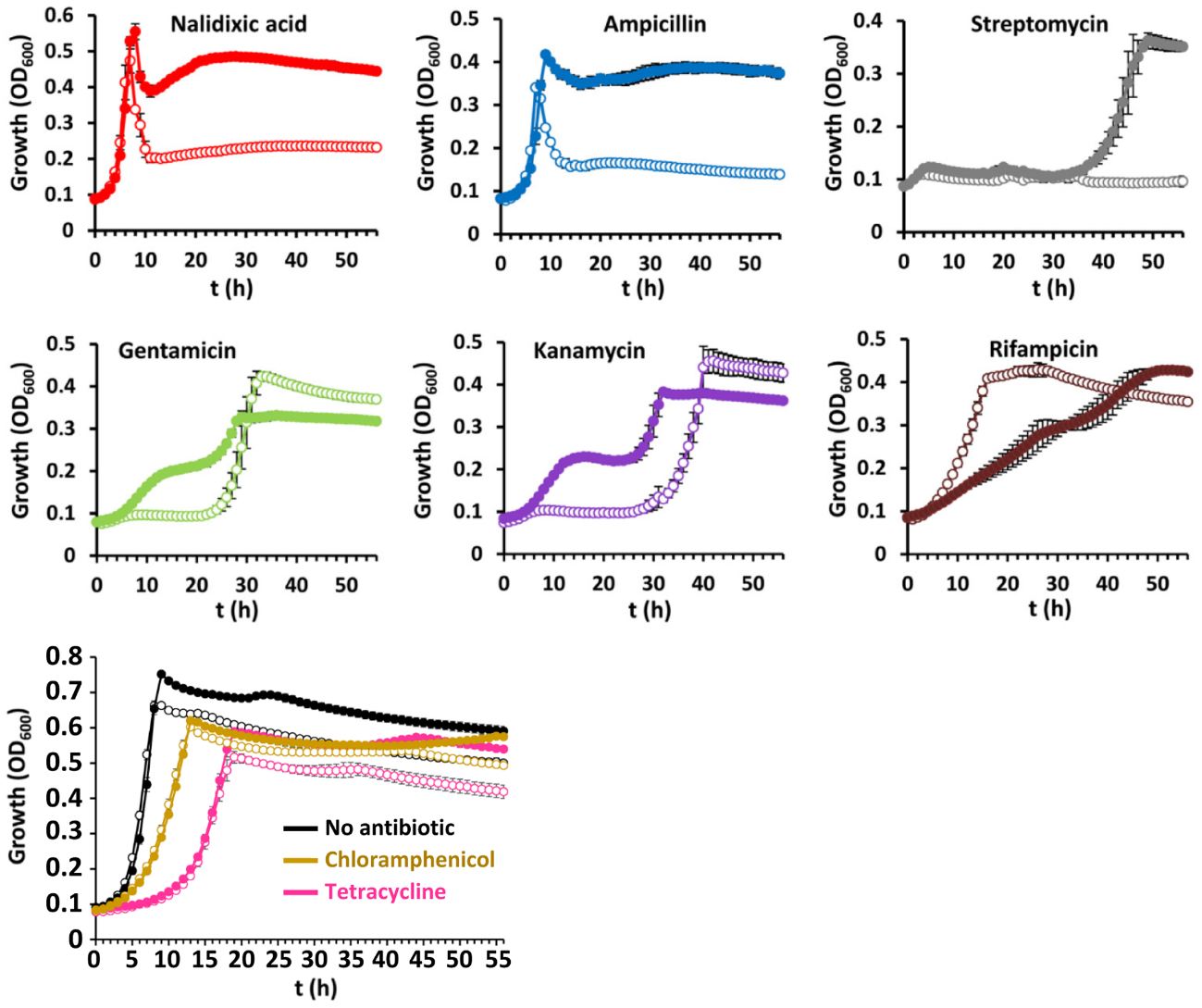


**Supplementary Figure S9. Effect of indole-3-acetic acid (IAA) on *Serratia plymuthica* growth in the presence of antibiotics with different mechanisms of action.** A153 was grown in minimal medium medium supplemented with 15 mM glucose in the absence (open symbols) and presence (filled symbols) of 1 mM IAA. Concentrations of antibiotics used: nalidixic acid, 1.3 µg/mL; ampicillin, 50 µg/mL; streptomycin, 25 µg/mL; gentamicin, 3.1 µg/mL; kanamycin, 12.5 µg/mL; rifampicin, 10 µg/mL; tetracycline, 1.5 µg/mL; chloramphenicol, 3.3 µg/mL. Growth was measured using Bioscreen Microbiological Growth Analyser (Oy Growth Curves Ab Ltd, Helsinki, Finland). Data are the means and standard deviations from five biological replicates. The assays were repeated three times and a representative assay is shown.

**
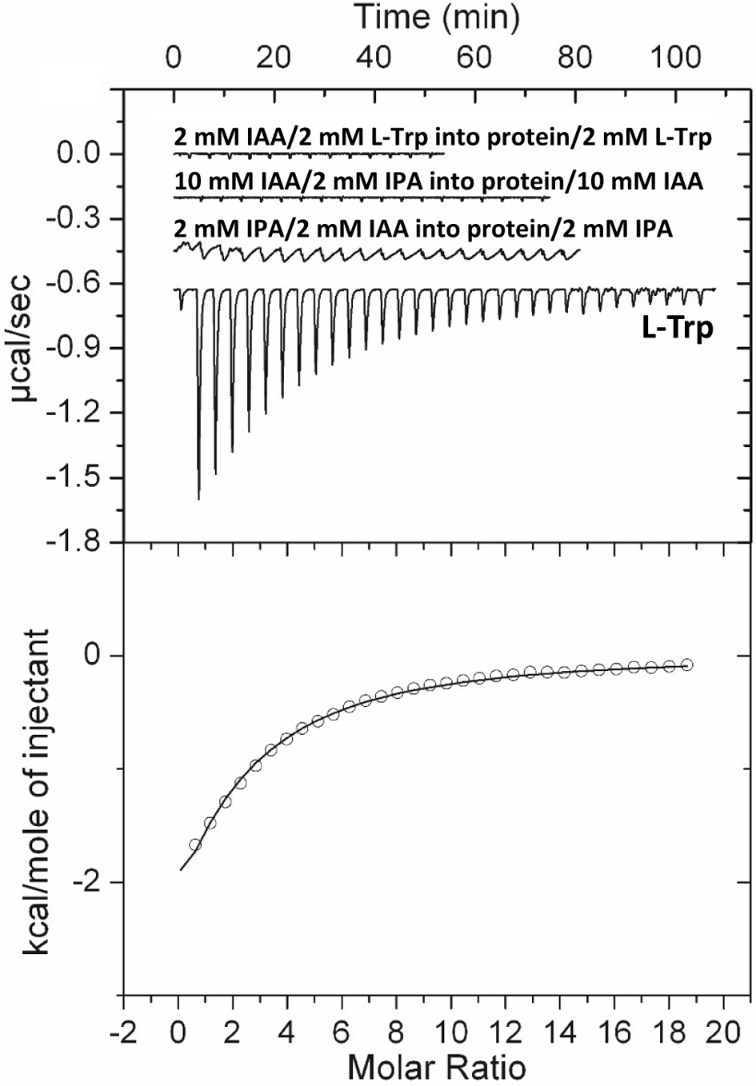
**

**Supplementary Figure S10. Isothermal titration calorimetry studies of the binding of different ligands to TrpR**_A153_ **of *Serratia plymuthica* A153.** Upper panel: Raw data for the titration of 25-50 μM of TrpR_A153_ (AWY96_RS12805) with 9.6 μL aliquots of 1-2 mM ligand solutions. Lower panel: Integrated, dilution heat-corrected and concentration-normalized peak areas fitted using the ‘One binding site’ model of the MicroCal version of ORIGIN. Symbols used in the lower panel are defined in the upper panel of this figure. L-Trp, L-tryptophan; IAA, indole-3-acetic acid; IPA, indole-3-pyruvic acid.

**References**

1. Woodcock DM, Crowther PJ, Doherty J, Jefferson S, DeCruz E, Noyer-Weidner M, Smith SS, Michael MZ, Graham MW. 1989. Quantitative evaluation of *Escherichia coli* host strains for tolerance to cytosine methylation in plasmid and phage recombinants. Nucleic Acids Res 17:3469–3478.

2. Herrero M, de Lorenzo V, Timmis KN. 1990. Transposon vectors containing non-antibiotic resistance selection markers for cloning and stable chromosomal insertion of foreign genes in gram-negative bacteria. J Bacteriol 172:6557–6567.

3. Jeong H, Barbe V, Lee CH, Vallenet D, Yu DS, Choi SH, Couloux A, Lee SW, Yoon SH, Cattolico L, Hur CG, Park HS, Segurens B, Kim SC, Oh TK, Lenski RE, Studier FW, Daegelen P, Kim JF. 2009. Genome sequences of *Escherichia coli* B strains REL606 and BL21(DE3). J Mol Biol 394:644–652.

4. Demarre G, Guerout AM, Matsumoto-Mashimo C, Rowe-Magnus DA, Marliere P, Mazel D. 2005. A new family of mobilizable suicide plasmids based on broad host range R388 plasmid (IncW) and RP4 plasmid (IncPalpha) conjugative machineries and their cognate *Escherichia coli* host strains. Res Microbiol 156:245–255.

5. Matilla MA, Leeper FJ, Salmond GP. 2015. Biosynthesis of the antifungal haterumalide, oocydin A, in *Serratia*, and its regulation by quorum sensing, RpoS and Hfq. Environ Microbiol 17:2993–3008.

6. Matilla MA, Nogellova V, Morel B, Krell T, Salmond GP. 2016. Biosynthesis of the acetyl-CoA carboxylase-inhibiting antibiotic, andrimid in *Serratia* is regulated by Hfq and the LysR-type transcriptional regulator, AdmX. Environ Microbiol 18:3635–3650.

7. Matilla MA, Salmond GP. 2014. Bacteriophage phiMAM1, a viunalikevirus, is a broad-host-range, high-efficiency generalized transducer that infects environmental and clinical isolates of the enterobacterial genera *Serratia* and *Kluyvera*. Appl Environ Microbiol 80:6446–6457.

8. Kaniga K, Delor I, Cornelis GR. 1991. A wide-host-range suicide vector for improving reverse genetics in gram-negative bacteria: inactivation of the *blaA* gene of *Yersinia enterocolitica*. Gene 109:137–141.

9. Dennis JJ, Zylstra GJ. 1998. Plasposons: modular self-cloning minitransposon derivatives for rapid genetic analysis of gram-negative bacterial genomes. Appl Environ Microbiol 64:2710–2715.
